# Derived alleles of two axis proteins affect meiotic traits in autotetraploid *Arabidopsis arenosa*

**DOI:** 10.1073/pnas.1919459117

**Published:** 2020-04-09

**Authors:** Chris Morgan, Huakun Zhang, Clare E. Henry, F. Chris H. Franklin, Kirsten Bomblies

**Affiliations:** ^a^Department of Cell and Developmental Biology, John Innes Centre, NR4 7UH Norwich, United Kingdom;; ^b^Key Laboratory of Molecular Epigenetics of Ministry of Education, Northeast Normal University, 130024 Changchun, China;; ^c^School of Biosciences, The University of Birmingham, B15 2TT Edgbaston, Birmingham, United Kingdom;; ^d^Institute of Molecular Plant Biology, Department of Biology, Swiss Federal Institute of Technology (ETH) Zürich, 8092 Zürich, Switzerland

**Keywords:** meiosis, polyploid, genome duplication, adaptation, evolution

## Abstract

Polyploidy, which results from whole genome duplication (WGD), has shaped the long-term evolution of eukaryotic genomes in all kingdoms. Polyploidy is also implicated in adaptation, domestication, and speciation. Yet when WGD newly occurs, the resulting neopolyploids face numerous challenges. A particularly pernicious problem is the segregation of multiple chromosome copies in meiosis. Evolution can overcome this challenge, likely through modification of chromosome pairing and recombination to prevent deleterious multivalent chromosome associations, but the molecular basis of this remains mysterious. We study mechanisms underlying evolutionary stabilization of polyploid meiosis using *Arabidopsis arenosa*, a relative of *A. thaliana* with natural diploid and meiotically stable autotetraploid populations. Here we investigate the effects of ancestral (diploid) versus derived (tetraploid) alleles of two genes, *ASY1* and *ASY3*, that were among several meiosis genes under selection in the tetraploid lineage. These genes encode interacting proteins critical for formation of meiotic chromosome axes, long linear multiprotein structures that form along sister chromatids in meiosis and are essential for recombination, chromosome segregation, and fertility. We show that derived alleles of both genes are associated with changes in meiosis, including reduced formation of multichromosome associations, reduced axis length, and a tendency to more rod-shaped bivalents in metaphase I. Thus, we conclude that ASY1 and ASY3 are components of a larger multigenic solution to polyploid meiosis in which individual genes have subtle effects. Our results are relevant for understanding polyploid evolution and more generally for understanding how meiotic traits can evolve when faced with challenges.

## Significance

Genome duplication is an important factor in the evolution of eukaryotic lineages, but it poses challenges for the regular segregation of chromosomes in meiosis and thus fertility. To survive, polyploid lineages must evolve to overcome initial challenges that accompany doubling the chromosome complement. Understanding how evolution can solve the challenge of segregating multiple homologous chromosomes promises fundamental insights into the mechanisms of genome maintenance and could open polyploidy as a crop improvement tool. We previously identified candidate genes for meiotic stabilization of *Arabidopsis arenosa*, which has natural diploid and tetraploid variants. Here we test the role that derived alleles of two genes under selection in tetraploid *A. arenosa* might have in meiotic stabilization in tetraploids.

Whole genome duplication, which results in polyploidy, increases genome complexity, and plays roles in speciation, adaptation, and domestication (1–5). Yet when polyploids are newly formed they face numerous challenges (1, 4, 6, 7); one of the biggest is the reliable segregation of the additional copies of each chromosome in meiosis (1, 7, 8). In diploids, each chromosome has just one homologous partner it can pair and recombine with in meiosis (9), but in polyploids more homologous partners are available, and this can result in multivalent associations, as well as unpaired univalents that indicate failures in pairing or recombination (1, 7, 8). Evolved polyploids rarely form multivalents or univalents, suggesting that preventing them is an important aspect of meiotic stability in polyploids (8, 10). The molecular basis of multivalent prevention and meiotic stabilization in polyploids remains almost entirely mysterious.

A major factor in the evolution of meiotic stability in polyploids seems to involve modulation of crossing over among homologous chromosomes (8). Solutions to polyploid meiosis fall into two major phenotypic groups that follow the distinction between allo- and autopolyploids. Allopolyploids have a hybrid origin and thus carry two or more at least partially distinct “subgenomes” (4, 11). Stable bivalent formation in allopolyploids involves strengthening pairing partner choice such that chromosomes recombine preferentially with partners from the same subgenome (6, 12). In contrast, autopolyploids arise from within-species genome duplications (4, 11), do not contain distinguishable subgenomes, and lack consistent pairing preferences (6–8, 13). The ability to primarily form bivalents in autopolyploids has been proposed to rely in large part on a reduction in crossover (CO) rates, ideally to one per chromosome, which at least in theory can suffice to prevent multivalent formation (8). Indeed, meiotically stable autopolyploids generally have low CO rates (10, 14, 15), and neopolyploid fertility negatively correlates with the diploid CO rate (16, 17). Evolved autopolyploids also usually have distal COs (8, 18–20); why this is important is less clear.

We use *Arabidopsis arenosa* as a model to understand the molecular basis of autopolyploid meiotic stabilization. This species is a close relative of *A. thaliana* with naturally occurring diploid and autotetraploid populations (21, 22). The tetraploid lineage arose just once, albeit with subsequent gene flow from diploids (23, 24). Meiosis in autotetraploid *A. arenosa* is stable, while that of neopolyploids is not, the latter being characterized by abundant multivalent associations, univalents, chromosome mis-segregation, and low fertility (14, 25). Meiosis in evolved tetraploid *A. arenosa* has several key features, including that COs are few in number, often just over one per bivalent, and that neotetraploids have considerably more multivalents (correlated with low fertility) than evolved tetraploids (14). To understand which genetic changes might be responsible for the evolution of these traits, we previously used genome scans of *A. arenosa* to identify loci that show strong evidence of having been targets of natural selection in tetraploids (14, 26). Among these are multiple genes encoding proteins important for meiotic processes such as cohesion, axis formation, synapsis, and homologous recombination.

In this study, we compare effects on tetraploid meiosis of derived (tetraploid) and ancestral (diploid) alleles of two of the genes that we previously found to be under selection in tetraploid *A. arenosa*, *ASYNAPSIS1* (*ASY1*) and *ASYNAPSIS3* (*ASY3*). ASY1 and ASY3 are homologs of the yeast axis proteins Hop1 and Red1, respectively (27, 28). The axes are protein structures that form along the lengths of replicated chromosomes in meiotic prophase I and are essential for chromosome pairing, synapsis, and homologous recombination (9). Mutants for *ASY1* or *ASY3* are defective in synapsis, have low CO rates, high univalent rates, and are nearly sterile (28, 29). Both proteins are also important in yeast for directing repair partner choice to the homolog rather than the sister chromatid, an important feature of meiotic recombination (30–33). Hop1 and Red1 interact directly in yeast (34–36), and this is functionally important for recombination and synapsis (35). Like their yeast counterparts, plant homologs also interact (28, 37). Thus, these proteins are good candidates for collaboratively causing the changes in the recombination rate and/or pattern that we see in tetraploids, which we test here using genetic and cytological approaches.

## Results

### Effects of Alternate *ASY1* Alleles on Metaphase I Phenotypes.

To begin testing the function of the derived alleles of the axis proteins in tetraploid *A. arenosa*, we took advantage of naturally segregating variation. While most *A. arenosa* populations carry only the tetraploid (*T*) allele of *ASY1*, we previously identified some tetraploid populations that segregate diploid (*D*) alleles of *ASY1* as rare variants (26). Thus, we generated a PCR marker to detect a ploidy-differentiated polymorphism (*Materials and Methods*) and used this to identify plants grown from seeds collected from Triberg, Germany (TBG), with the genotype *ASY1*-*TTTD* (carrying three copies of the *T* allele and one of the *D* allele). We intercrossed these and bred them to ultimately generate F_2_ populations segregating individuals homozygous for either allele at *ASY1*. We previously showed that the *D* and *T* alleles differed by five amino acids (38). By isolating and sequencing complementary DNAs (cDNAs) from *D/T* heterozygous tetraploids and diploids, we confirmed that naturally segregating *D* and *T* alleles carry these polymorphisms (*SI Appendix*, Fig. S1); the functions of these amino acids are not known.

**Fig. 1.**
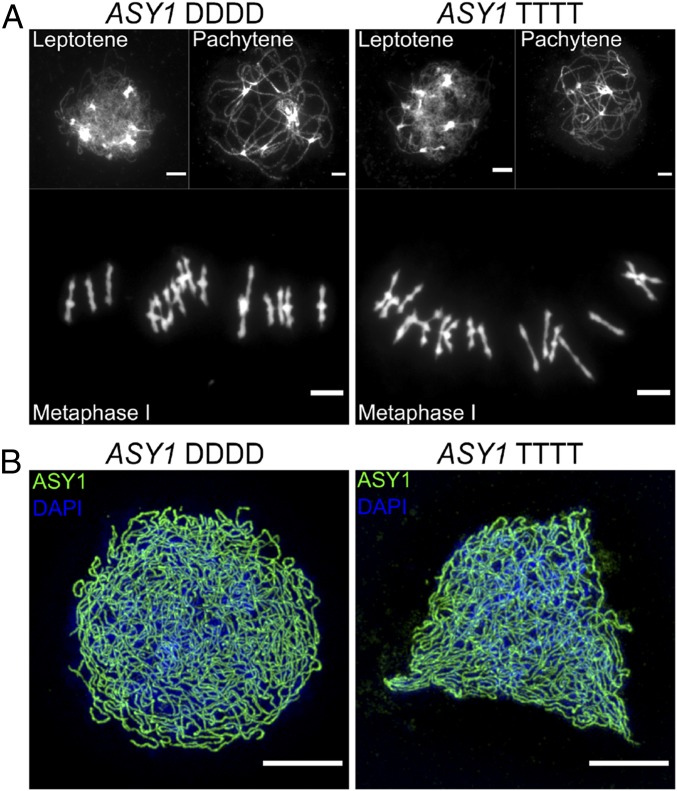
ASY1 diploid allele has normal meiosis in tetraploids. (*A*) DAPI-stained chromosomal spreads of leptotene, pachytene, and metaphase I cells from ASY1-DDDD and ASY1-TTTT plants. No obvious differences among genotypes were seen. (*B*) Leptotene cells labeled for ASY1 (green) and DAPI (blue) and imaged with 3D-SIM from ASY1-DDDD and ASY1-TTTT plants showing that ASY1 protein localizes normally along chromosome axes in both genotypes. (Scale bars, 5 µm.)

In our segregating populations, we denote the alternate homozygous genotypes *ASY1-DDDD* and *ASY1-TTTT*. Heterozygotes in tetraploids can come in three forms (*DDDT*, *DDTT*, *TTTD*), which our marker cannot reliably distinguish, so we grouped these under the label “*TxD*.” We then studied individuals for differences in meiosis using cytology. Prophase I and metaphase I meiocytes stained with DAPI (to mark chromatin) or immunolabeled for ASY1 (to mark the axes in prophase I) from *ASY1-DDDD* and *ASY1-TTTT* plants showed that meiosis was qualitatively normal in both genotypes (Fig. 1), demonstrating that the ancestral *ASY1-D* allele is functional in the tetraploid context.

**Fig. 2.**
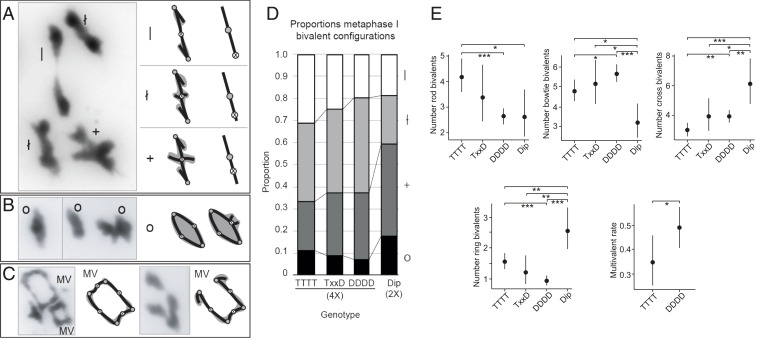
Metaphase I bivalent shapes. (*A*) Single crossover bivalents with little (|), intermediate (ł), or extensive (+) chromatin beyond a chiasma. (*Left*) Examples from metaphase spreads. (*Right*) Diagrammatic interpretations with stick interpretations and chromatin shaded in gray. CO position interpretation is shown in rightmost cartoons. Shaded circles indicate centromeres, and open circles with an “X” indicate positions of crossovers. (*B*) Examples of two-chiasma “ring” configurations (o) and diagrammatic interpretations. (*C*) Examples of ring quadrivalents (*Left*) and a chain quadrivalent (*Right*) with four and three chiasmata, respectively, along with diagrammatic interpretations. (*D*) Stacked bar graph showing metaphase I bivalent configurations (rod, |; bowtie, ł; cross, +; ring, O) as a proportion of scorable bivalents in diploid (Dip, 2×) and tetraploid (4×) *A. arenosa* with different genotypes at ASY1, where DDDD = homozygous for diploid allele, TxxD = heterozygous (TDDD, TTDD, or TTTD), and TTTT = homozygous for tetraploid allele. Values are calculated as means per genotype from bivalent proportions given in *SI Appendix*, Table S1. Shading indicates bivalent shapes as indicated to the right of the graph. Chiasma configuration interpretations are based on those in ref. 39. (*E*) Bivalent shape numbers and multivalent (MV)-containing cell proportions in diploids and ASY1-segregating tetraploid lines. Note that diploid counts have been doubled to enable direct comparison with tetraploid counts. Dots indicate trait means and error bars 95% confidence intervals calculated from GLMM models (*SI Appendix*, Table S3) from data in *SI Appendix*, Table S1. *P* values are indicated by bars above each graph: *P < 0.05, **P < 0.005, and ***P < 0.0005 (from *SI Appendix*, Table S3).

Bivalent shapes in metaphase I spreads are sometimes used to assess approximately where chiasmata are located (cytologically visible connections among chromosomes that are the outcomes of CO events) and how many there are (39) (Fig. 2 *A* and *B*). Metaphase I spreads also allow us to quantify multivalent formation rates (Fig. 2*C*). We measured all traits “blind” on metaphase spreads; all genotype information was temporarily removed and random numbers were assigned to images to prevent inadvertent biases in our phenotypic assessments. To test whether the *D* vs. *T* alleles of *ASY1* are associated with quantitative differences in meiosis, we first examined metaphase I spreads from developing anthers of *ASY1-DDDD*, *ASY1-TTTT*, and *ASY1-TxD* plants. We also sampled a diploid from a population from Streçno, Slovakia (SN), that is, within our sampling, the closest relative of the tetraploid (23). For each cell, we counted all bivalent types described in Fig. 2 *A*–*C*. For the diploid (Dip, 2×), we sampled 25 images from one plant (total bivalents scored = 182). Among tetraploids, we included only images that were of sufficient quality that 10 or more bivalents of the 16 possible could be scored per image. For the *DDDD* genotype, we sampled 170 images from eight individuals (total bivalents scored = 2,266). For the *TTTT* genotype, we sampled 100 images from four individuals (total bivalents scored = 1,363). For *TxD*, we sampled 25 images from one plant (total bivalents scored = 341). All assayed trait values are given in *SI Appendix*, Table S1.

As a first approach to determine if there were differences in the frequency of different bivalent categories (rod “|”, bowtie “†”, cross “+”, ring “O” in Fig. 2 *A*–*C*) in metaphase I cells for the *ASY1* segregants, we performed χ^2^ tests using bivalent count data per cell from each genotype to determine if we could reject the null hypothesis (H_0_) that bivalent distribution among categories does not differ between genotypes. This analysis showed there are significant differences among genotypes in bivalent shape distribution (χ^2^ test, *P* = 2.2 × 10^−16^, ϕc = 0.12; *SI Appendix*, Table S2). We then used post hoc pairwise χ^2^ tests with Bonferroni correction to determine if significant differences occurred between individual pairs of genotypes (*SI Appendix*, Table S2). Count data from diploid cells were doubled to enable comparison with tetraploid cells. From this analysis, we found that the homozygous genotypes (*ASY1-DDDD* and *ASY1-TTTT*) differed significantly in bivalent shape distributions (Fig. 2 *D* and *E*; χ^2^ test, *P* = 3.67 × 10^−7^, ϕc = 0.15; *SI Appendix*, Table S2), and both homozygotes differed from the diploid (χ^2^ test, *P* = 1.83 × 10^−9^, ϕc = 0.2, and *P* = 1.15 × 10^−13^, ϕc = 0.25, respectively).

Due to the pooling of data from multiple plants across two experiments, this first statistical approach is vulnerable to type I errors as a result of sacrificial pseudoreplication (i.e., individual plants may bias the results due to biological variation within individuals of the same genotype). We therefore extended our statistical analysis using a Poisson generalized linear mixed model (Poisson-GLMM), which is well suited for analyzing count data, to analyze the bivalent shape counts for each cell. GLMM analysis also showed that bivalent shape distributions differ significantly between diploids and tetraploids as well as between *ASY1-DDDD* and *ASY1-TTTT* plants (*SI Appendix*, Table S3).

From genotype means calculated in GLMM analyses, we found that the diploid had fewer “|” bivalents than *ASY1-TTTT* tetraploids (Poisson-GLMM, 2.63 SE = [+0.50, −0.42] vs. 4.21 SE = [+0.35, −0.32]; *P* = 0.014). Similarly, tetraploid *ASY1-DDDD* individuals also had significantly fewer “|” bivalents than *ASY1-TTTT* plants (Poisson-GLMM, 2.63 SE = [+0.18, −0.17] vs. 4.21 SE = [+0.35, −0.32]; *P* = 4.4 × 10^−6^; Fig. 2*E* and *SI Appendix*, Table S3). Conversely, both the diploid and *ASY1-DDDD* tetraploids had significantly more “+” bivalents than *ASY1-TTTT* plants (*SI Appendix*, Table S3). Heterozygotes (*ASY1-TxD*) were intermediate between *ASY1-DDDD* and *ASY1-TTTT*, but did not differ significantly from either (Fig. 2*E* and *SI Appendix*, Table S2).

To determine if there were differences in the frequency of multivalent occurrence within metaphase I cells (yes/no data indicate presence or absence of at least one [and usually only one] multivalent in metaphase I cells; *SI Appendix*, Table S1), we used Fisher’s exact test, as well as a binomial generalized linear mixed model (Binomial-GLMM). We calculated differences in multivalent occurrence between the *ASY1-DDDD* and *ASY1-TTTT* genotypes only. Both types of analysis showed that the *ASY1-DDDD* plants had a significantly higher proportion of cells with multivalents than *ASY1-TTTT* plants (Binomial-GLMM, 0.49 SE [+0.043, −0.043] vs. 0.35 SE [+0.055, −0.051], *P* = 0.042; Fisher’s exact test *P* = 0.031, ϕc = 0.14; Fig. 2*E* and *SI Appendix*, Table S3).

### Effects of Both *ASY1* and *ASY3 D* and *T* Alleles on Metaphase I Phenotypes.

A critical partner for ASY1, the axis protein ASY3 (28, 35), also shows strong evidence of selection in *A. arenosa* (14). As for *ASY1*, we also previously described *ASY3* sequences from diploid and tetraploid *A. arenosa* in detail (38). Short read alignments (24, 38) showed that the diploid (*D*) and tetraploid (*T*) alleles differ by 18 amino acids, all of which we confirmed with cDNA sequencing of *ASY3-D* and *ASY3-T* alleles (*SI Appendix*, Fig. S2). In addition, we identified five additional amino acid differences between the *D* and *T* alleles as well as a duplication of 26 amino acids not represented in the *ASY3* short read alignments. The duplicated region had two amino acid differences that led to the tetraploid allele having two predicted SUMOylation sites in this region, and the diploid none (*SI Appendix*, Fig. S2). This is intriguing in light of previous work showing that the yeast ASY3 homolog Red1 is SUMOylated during meiosis (40).

Since both ASY1 and ASY3 are axis components, we wanted to test whether derived (*T*) alleles of *ASY1* and *ASY3* affect the same phenotypes. We used a different strategy to generate segregating populations for both genes, as we did not find naturally segregating *ASY3 D* alleles in the tetraploid plants that we tested (*Methods*). In brief, we used colchicine doubled diploids to generate neotetraploids (*DDDD* at both *ASY1* and *ASY3*) that we then backcrossed twice to established (natural) TBG tetraploids (*TTTT* for most genes). We then intercrossed BC2 progeny, identified *ASY1-D* and *T* carriers and *ASY3-D* and *T* carriers, and bred plants through to the F_3_ to generate segregating populations for both *ASY3-D* and *ASY3-T*. We designated F_3_ genotypes with a shorthand DD, DT, TD, or TT to indicate their homozygous genotypes at *ASY1* and *ASY3*, respectively; for example, a “DT” plant is *DDDD* at *ASY1* and *TTTT* at *ASY3*. We did two runs of the experiment, which for analysis were combined together (data are given in *SI Appendix*, Tables S4 and S5). In total, for DD, we scored 73 images from 7 plants (total bivalents scored = 928); for DT, we scored 114 images from 10 plants (total bivalents scored = 1,459); for TD, we scored 105 images from 9 plants (total bivalents scored = 1,353); and for TT, we scored 121 images for 10 plants (total bivalents scored = 1,551). As above, we included only spreads of sufficient quality that at least 10 of the 16 bivalents could be scored. See *SI Appendix*, Tables S4 and S5, for details and data.

**Fig. 3.**
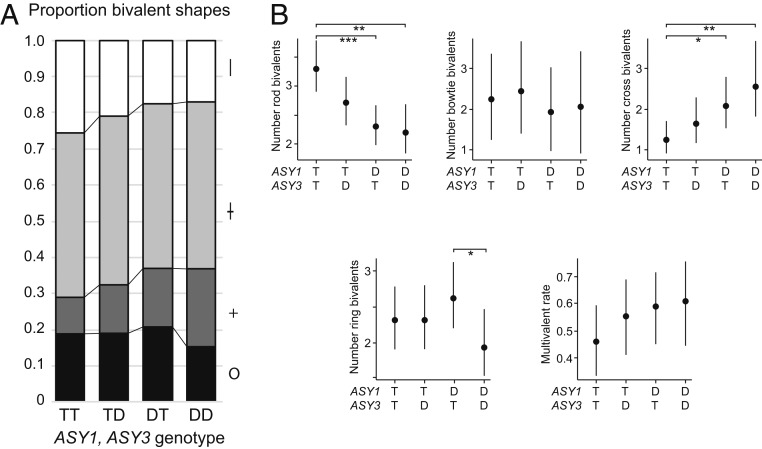
Associations of ASY1 and ASY3 genotype with metaphase bivalent shape. (*A*) Stacked bar graph showing, as in Fig. 2*D*, proportions of different bivalent shapes (as proportion of all scorable bivalents: rod, |; bowtie, ł; cross, +; ring, O) for the ASY1/ASY3-segregating population means calculated for each genotype from proportions calculated in *SI Appendix*, Table S5. Genotypes: DD, homozygous for D alleles at both genes; DT, homozygous D for ASY1, homozygous T at ASY3; TD, homozygous T for ASY1, homozygous D at ASY3; TT, homozygous T for both genes. (*B*) Bivalent shape numbers per cell and proportion MV-containing cell proportions in ASY1/ASY3-segregating tetraploid lines. Dots indicate trait means and error bars 95% confidence intervals calculated from GLMM models (*SI Appendix*, Table S7) from data in *SI Appendix*, Tables S4 and S5. *P* values are indicated by bars above each graph: *P < 0.05, **P < 0.005, and ***P < 0.0005 (from *SI Appendix*, Table S7).

We phenotyped all genotypes using the same cytological methods described above. Again, we scored all images blind to genotype. As for the ASY1 experiment described above, we first used χ^2^ tests using bivalent count data to determine if bivalent shape distribution differs between genotypes (it does; χ^2^ test, *P* = 5.82 × 10^−16^, ϕc = 0.08; *SI Appendix*, Table S6) and used post hoc pairwise χ^2^ tests with Bonferroni correction to determine if significant differences occurred between individual pairs of genotypes (*SI Appendix*, Table S6). Using χ^2^ analyses, all genotypes except DT and TD differed from each other significantly for bivalent shape distribution, suggesting that both *ASY1* and *ASY3* allele states affect this trait (χ^2^ test *P* = 8.94 × 10^−3^, ϕc = 0.07 for TD vs. TT; *P* = 4.60 × 10^−15^, ϕc = 0.17, for DD vs. TT; *SI Appendix*, Table S6). We also used Poisson-GLMM to analyze bivalent count data as for the ASY1 experiment above (*SI Appendix*, Table S7). In this analysis, TT differed significantly from both DD and DT for rod “|” and cross “+” bivalents. TD plants also had fewer rod bivalents per cell than TT plants at a level that was approaching significance (Poisson-GLMM, 2.70 SE [+0.22, −0.20] vs. 3.29 SE [+0.23, −0.22], *P* = 0.0541), suggesting that the *T* allele of *ASY3* may have a similar, albeit modest, effect on bivalent shape as the *ASY1 T* allele. Trait means suggest that *ASY1* and *ASY3* trend in the same direction and, in the case of *ASY1*, also in the same direction as the ASY1 experiment above (Fig. 3 and *SI Appendix*, Table S7). The only other significant trend was that DD and DT differed for ring bivalent frequency (Poisson-GLMM, 1.95 SE [+0.24, −0.22] vs. 2.62 SE [+0.25, −0.23], *P* = 0.044; *SI Appendix*, Table S7). For multivalent rates, TT has the lowest proportion of cells with multivalents (Binomial-GLMM, 0.461 SE [+0.066, −0.065] vs. 0.61 SE [+0.076, −0.082] for DD; Fig. 3*B* and *SI Appendix*, Table S7), but although the difference is of about the same magnitude as in the ASY1 experiment, perhaps due to high variability between plants (Fig. 3*B*), the difference is not significant in either χ^2^ or Binomial GLMM analysis (*SI Appendix*, Tables S6 and S7).

To determine if the effect of *ASY1* and *ASY3* alleles on bivalent shapes was additive, or if there was evidence of an interaction effect between the genes, we fitted Poisson GLMM to the rod-bivalent and cross-bivalent count data using the ASY1 allelic state and the ASY3 allelic state as separate fixed factors. We then compared additive Poisson-GLMM models with Poisson-GLMM models that contained an ASY1:ASY3 interaction term. For both rod- and cross-bivalent datasets, additive models were the most parsimonious (with *ASY1* having a stronger effect than *ASY3*).

#### Prophase I chromosome length, CO rates, and positions.

**Fig. 4.**
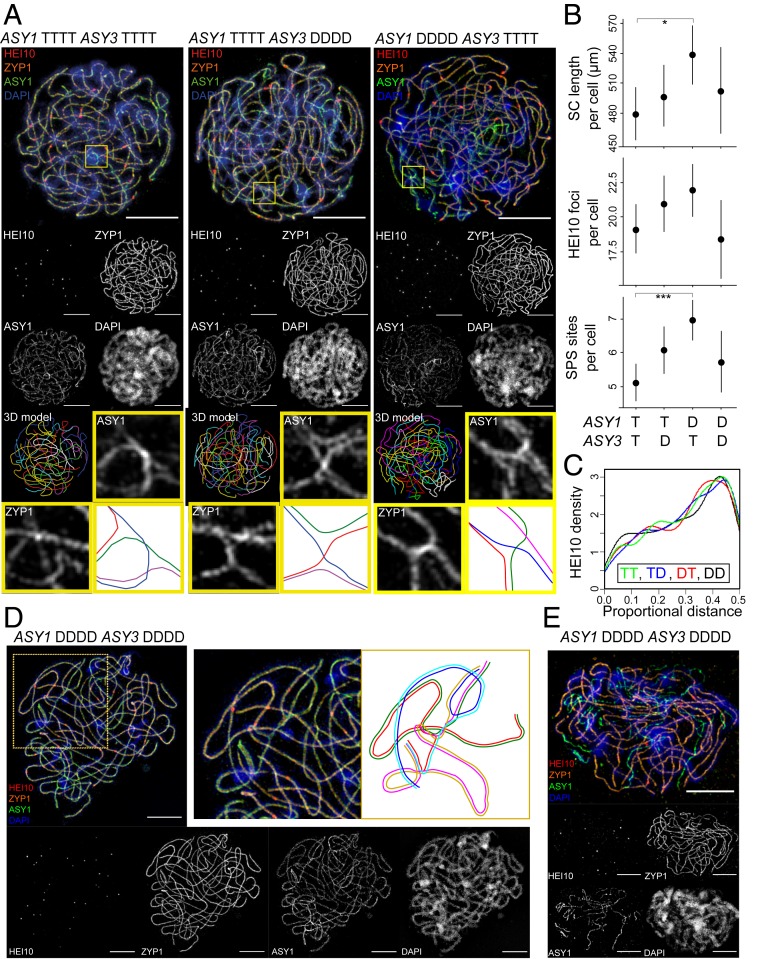
Immunocytological investigation of pachytene cells in DD, DT, TD, and TT genotypes generated from F_2_ backcrosses. (*A*) Representative pachytene cells from DT, TD, and TT individuals labeled for HEI10 (red), ZYP1 (orange), ASY1 (green), and DAPI (blue) and imaged with 3D-SIM. A 3D model of traced synapsed chromosomes is also shown for each cell. A region from each cell containing a synaptic partner switch has also been highlighted (yellow box), and enlarged images of these regions are shown alongside a cartoon representation of homolog interactions (axes of different chromosomes are shown in different colors) and synaptonemal complex localization (shaded orange) within the synaptic partner switch sites. (*B*) Plots comparing SC length per cell, late-HEI10 frequency per cell, and SPS site frequency per cell in DD, DT, TD, and TT *A. arenosa.* Plots show the estimated means from four to five biological replicates of genotypes DT, TD, and TT and two replicates from genotype DD. Error bars indicate ± 95% confidence intervals. Statistically significant differences are indicated by bars above the graphs (*P < 0.05, ***P < 0.0005, nested ANOVA). (*C*) Combined kernel density plot showing the distribution of single HEI10 focus positions from the nearest chromosome end as a proportion of total chromosome length for each genotype. (*D* and *E*) Pachytene cells from different DD individuals exhibiting unusual synaptic behavior. (*D*) A representative pachytene cell from one DD individual is shown with a zoomed-in region and accompanying cartoon depiction of the complex pattern of synaptic exchange that is occurring between six component chromosomes in this region (each component chromosome is labeled in a different color in the cartoon). (*E*) A representative pachytene cell from another DD individual in which extensive regions of asynapsis can be identified by the presence of long linear strands of brightly staining ASY1 signal that do not overlap with accompanying ZYP1 signal. (Scale bars, 5 µm.)

Metaphase I is a late stage in meiosis I that provides a “readout” of earlier events in prophase I in which ASY1 and ASY3 are active. Thus, we performed immunocytology on prophase I meiocytes from DD, DT, TD, and TT plants. We detected synaptonemal complex (SC) using an antibody against ZYP1, a core SC component (41), and used this signal to measure physical chromosome length. We counted and localized CO events using antibodies against HEI10, which, late in prophase I, marks sites destined to become COs (42). We measured all traits in cells from four to five individuals for each genotype (DD, DT, TD and TT; see Fig. 4 for representative examples and *SI Appendix*, Table S8, for trait values). We found that DT individuals had a significantly longer SC length per cell than TT individuals (unweighted means, DT = 538 µm, TT = 480 µm, nested ANOVA F_1,7_ = 8.6360, *P* = 0.0260; *SI Appendix*, Table S8). No other between-group differences were significant, including in the distribution of single HEI10 focus positions from the nearest chromosome end (Fig. 4*C*), although the TT genotype had the lowest unweighted mean values for SC length per cell, late-HEI10 foci number per cell, and late-HEI10 focus distance from the nearest chromosome end when compared with either the DT or TD genotypes (Fig. 4*B* and *SI Appendix*, Table S8).

To test for any differences in the relationship between CO number per cell and total SC length per cell in different genotypes, we fitted regression lines to plots comparing CO number and SC length for each genotype (*SI Appendix*, Fig. S3) and compared these using analysis of covariance. No significant differences were detected in the slope of the regression lines between genotypes (*P* = 0.088).

In tetraploids, the multiple homologs present can all interact and sometimes pair with different partners along their lengths. This can result in “synaptic partner switch” (SPS) sites in prophase I that are clearly identifiable in three-dimensional structured illumination microscopy (3D-SIM) images (Fig. 4*A*). We quantified the number of SPS sites in the different genotypes and found that TT individuals have significantly fewer SPS sites per cell than the DT individuals (unweighted means, DT = 6.9, TT = 5.2, nested ANOVA F_1,5_ = 118.9246, *P =* 0.000113; Fig. 4*B* and *SI Appendix*, Table S8), showing that the *ASY1* allelic state affects this trait, at least when *ASY3* is *TTTT*. *ASY3* did not have a statistically significant effect (TD vs. TT; Fig. 4*B* and *SI Appendix*, Table S8). SPS sites are not merely a prophase I oddity: With CO placement on either side of an SPS, multichromosome associations can lead to persistent metaphase multivalents (see *SI Appendix*, Fig. S4, for explanation). We used the position of late-HEI10 foci relative to SPS sites (either on the same side or on opposite sides of an SPS; see *SI Appendix*, Fig. S4*B* for explanation) to predict which multichromosome groups would give rise to pairs of bivalents versus multivalents. We found that a higher proportion of the observed SPS-containing multichromosome groups in DT and TD are predicted to give rise to multivalents than in TT plants (*SI Appendix*, Fig. S5), consistent with the metaphase I data suggesting that at least *ASY1-T* is associated with lower multivalent rates.

For the comparison with DD, our analyses had low statistical power because only two of the four individuals sampled could be included due to more severe defects in two of them (see next section). However, there may be another factor as well. Considering the two traits (SC length and SPS sites per cell) where DT plants have significantly higher trait values to TT (Fig. 4*B*), suggesting an effect for *ASY1*, DD plants also have somewhat higher values (although not significantly so) relative to TT. Thus, either the lack of an overall significant trend is due to low power of the DD comparison, or perhaps the DT class truly is different. This could occur if this is an “out of context” effect; that is, the effect of *ASY1-T* is different if it is found together with the *T* allele of *ASY3*, than when it is paired with the *D* allele.

#### Prophase I defects in ASY1-DDDD/ASY3-DDDD plants.

From four DD individuals analyzed, it was possible to obtain quantitative measurements from pachytene cells for only two. The other two had severe but distinct defects in synapsis that prevented paths of all component chromosomes from being accurately traced. In one DD individual (plant 1T-37), at least one synaptic exchange was observed among more than four component chromosomes (Fig. 4*C*) in all cells imaged (*n* = 17), a phenotype not observed in any other individual in the experiment. At metaphase, this plant did not show more multivalents or univalents than others (two-tailed, unequal variance *t*-test *P* values 0.21 to 0.82), suggesting that abnormal prophase associations are resolved prior to metaphase. In a second DD individual (plant 64), we did not see evidence of nonhomologous exchange, but pachytene cells failed to complete synapsis (Fig. 4*D*) in all cells imaged (*n* = 9). The stage was identified as late pachytene (rather than, e.g., zygotene, where asynapsis would be expected) by the presence of bright HEI0 foci that overlapped with SC, a stage at which SC was fully polymerized in all other individuals. This plant had more univalents in metaphase I than any other plant sampled, so we did not include it in the analyses above (all other DD plants, *n* = 37 metaphase I spreads, average univalent per cell = 0.51; plant 64, *n* = 62 metaphase I spreads, average univalent per cell = 1.13; two-tailed, unequal variance *t*-test *P* value = 0.013). Since the defects in the two plants are distinct and do not appear in all DD plants, we speculate that they do not arise directly from allelic variation at *ASY1* and *ASY3*, suggesting that the DD genotype creates a genetic context that makes plants especially sensitive to the segregation of additional meiosis genes, compared to those with *T* alleles at either *ASY1* or *ASY3*.

## Conclusions

Genes encoding two interacting meiotic chromosome axis proteins were among the strongest genome-wide outliers for evidence of selection during evolution of tetraploid *A. arenosa*, suggesting that they might play a role in meiotic stabilization of the polyploid lineage (14, 26). Here we test the functional consequences of their divergence and show that derived alleles of *ASY1* and to a lesser extent *ASY3*, are associated with subtle effects on several key features that are likely important for stable tetraploid meiosis: 1) a reduction in multivalent formation rates, 2) a trend toward more “rod-shaped” bivalents in metaphase that could reflect increased chromatin condensation, and 3) a reduction in the length of the chromosome axes.

The chromosome axes provide a structural context for meiotic events including homologous recombination and synapsis (9). ASY1 and ASY3 are major components of the axes in plants and, like their counterparts in other eukaryotes, have important roles in chromosome pairing and recombination (28, 43, 44). In yeast, the homologs of ASY1 and ASY3, Hop1 and Red1, can affect CO number and mediate homolog bias during repair of double-strand break (DSB) events (30, 32, 33, 45–47). In *A. thaliana*, *asy1* and *asy3* mutants have a sharp reduction in recombination rate (28, 30, 46). Based on their roles in pairing and recombination, we initially hypothesized that the derived alleles of ASY1 and ASY3 might be responsible for the lower cross-over number in tetraploid *A. arenosa* and thereby prevent deleterious multivalent formation since a CO number reduction to one per bivalent in theory could suffice to ensure that only bivalents form in meiosis (8). However, counting HEI10 foci (a marker of class I COs in late prophase I), we did not find evidence in this experiment that the derived *T* alleles of either ASY1 or ASY3 significantly affect per-chromosome or genome-wide CO rates.

Derived alleles of ASY1 and, to a lesser extent, ASY3 did associate with an increased number of bivalents with “rod-like” shapes in metaphase I spreads, which are sometimes seen as suggestive of more distal CO positions in metaphase I spreads (39). A role for axis proteins in generating more distal cross-over placement is at face plausible: In *A. thaliana*, *asy1* and *asy3* mutants have very few cross-overs, but those that remain are primarily subtelomeric (27, 39). This may be related to the observation that telomeres cluster in *A. thaliana*, and this clustering is largely maintained in the *asy1* mutant, perhaps allowing interhomolog recombination events to progress in these regions due to the proximity of the chromosomes (43). Telomeres also cluster in *A. arenosa* early in meiosis (25). However, the idea that chiasmata are more distal in *T* allele carriers of *ASY1* or *ASY3* is not supported when measuring HEI10 focus position relative to chromosome ends in prophase I. The discrepancy between the metaphase I interpretation and the prophase I data suggests that the difference in bivalent shapes seen among genotypes in metaphase I is likely not a consequence of CO positioning, but reflects some other feature such as differences in chromatin compaction or bivalent distortion. For example, strong condensation of chromatin at chromosome tips has been previously suggested as a reason for the observation of apparently terminal metaphase I chiasmata in rye (48). It could be that the T alleles of the axis proteins promote a similar fate in *A. arenosa*, but this remains to be tested. By which mechanism the *T* alleles of the *A. arenosa* axis proteins cause differences in bivalent shape observed in metaphase I, and whether these shape changes are directly beneficial to polyploid meiosis or a by-product of another feature that is beneficial, remains to be tested.

Plants homozygous for *ASY1-T* alleles had significantly fewer cells with multivalents in metaphase I than *ASY1-*D homozygotes, suggesting that the *ASY1 T* alleles may contribute to this important aspect of polyploid meiotic stabilization (8). This is corroborated by the observation that, in prophase I, *ASY1-TTTT* plants have fewer synaptic partner switches (partner switches are a prerequisite for multivalent formation), and among those multichromosome associations that they do make in prophase I, a lower proportion of those configurations are predicted to give rise to multivalents. The results from prophase I suggest that the reduction in multivalent formation in the presence of the *ASY1-T* allele may at least in part be due to a stronger preference for COs to be placed on the same side relative to partner switch sites, which we previously proposed as an important feature of multivalent prevention in polyploids because this geometry leads to dissolution into a pair of bivalents (8). The observation that the *ASY1* allele state may also influence axis length supports the idea that allelic variation at *ASY1* can alter axis organization in some way, and it may be that this somehow promotes the “safer” positioning of CO sites on the same side of partner switch sites. How exactly the derived changes in ASY1, or perhaps in both of these axis proteins, can accomplish this, remains an exciting future research question.

In yeast and plants, extension of Hop1/ASY1 along axes depends on Red1/ASY3, but not the reverse (28, 49). The interaction of Red1 and Hop1 is important not only for localization, but also for crossing over and synapsis; a single mutation in Red1 that disrupts its interaction with Hop1 disrupts both processes (35). Moreover, Hop1 interacts with Holliday junctions during recombination maturation, and this interaction, which is likely important for stabilizing recombination intermediates, is potentiated by Red1 (50, 51). Thus, we initially hypothesized that ASY1 and ASY3 might have coevolved and have synergistic effects in tetraploid *A. arenosa*. Our GLMM analyses, however, suggest that the effects of the derived alleles of the two genes are primarily additive. Understanding the details of the interactions of *ASY1* and *ASY3* ancestral vs. derived alleles in diploid versus tetraploid *A. arenosa* to fully establish whether any change in the interaction between the two proteins affects the traits that we see remains an open question. The possibility that DT and TD plants might have more extreme phenotypes relative to TT and DD merits further exploration, as that possibility, too, could hint that the proteins have coevolved and have distinct effects when paired with the noncoevolved allele as might occasionally occur in nature (26).

Interestingly, our work is not alone in implicating either ASY1 or the axes in polyploid meiosis: In allohexaploid bread wheat, reducing expression of *ASY1* by RNA interference disrupts preferential pairing, causing chromosomes to associate also with homeologous partners (52). Moreover, absence of a locus (*Ph1*) that stabilizes preferential pairing in wheat (53) causes transcriptional up-regulation of *ASY1*, which is associated with increased homeologous pairing. That either increased or decreased expression of *ASY1* is associated with polyploid chromosome pairing aberrations in wheat (52) hints that proper axis function depends on dosage balance of ASY1 with other proteins, likely including its partner ASY3. The implication of ASY1 in the stabilization of allohexaploid wheat (52), as well as in the evolutionary stabilization of autopolyploid meiosis in *A. arenosa*, suggests that modification of the meiotic axes may play a role in the evolution of meiotic stabilization in polyploids more broadly.

Overall, the effects on meiosis that we see associated with derived versus ancestral alleles of *ASY1* and *ASY3* in autotetraploid *A. arenosa* are subtle. This fits with these alleles not being the sole “major effect” loci in polyploid stabilization, but two of a larger set of genes encoding interacting proteins under selection in the autotetraploid lineage (14, 26, 38). Thus, we believe that multiple genes evolved relatively subtle changes in order to alter the system without negatively affecting its essential functions. The chromosome axis appears to be part of this puzzle, but additional players such as the cohesin complex, the synaptonemal complex, and chromatin remodelers are almost certainly also involved (14, 26, 38), and it may be that the full difference between diploid and polyploid meiosis can be recapitulated only when the entire set is in one allele state or another. This type of pattern may be common when conserved and constrained cellular processes have to evolve to new states without disrupting their core functions in the process.

## Materials and Methods

### Plant Materials.

For diploid *A. arenosa*, we used the SN accession and for the tetraploid we used TBG (previously described in ref. 26). To generate lines segregating for ASY1 D vs. T alleles, we genotyped TBG plants using a PCR marker (see below) to identify carriers of diploid alleles. We then intercrossed these, generated F_1_’s, from which we identified suspected TTDD plants based on relative band intensities on agarose gels, and intercrossed these to generate F_2_ populations segregating homozygotes. To generate lines segregating for both *ASY1* and *ASY3 D* and *T* alleles, we generated tetraploid plants from diploid SN plants using colchicine (54). Once we confirmed plants using flow cytometry and cytology as fully tetraploid, we backcrossed these twice to TBG and then intercrossed the resulting plants to generate F_2_ populations segregating both genes. We grew plants in controlled environment rooms with 16 h of light (125 mMol cool white) at 20 °C and 8 h of dark at 16 °C.

### PCR Genotyping of ASY1 and ASY3.

We extracted DNA from plants using the MasterPure Complete DNA Purification Kit (Epicentre, Madison, WI). To genotype plants for ASY1 diploid (D) versus tetraploid (T) alleles, we first amplified an ∼300-bp fragment with primers F1 (5′-TTT​GGT​TTT​CGT​TTT​GCT​GA-3′) and R1 (5′- GAG​ATT​CAG​CGT​CCA​TAG​GC-3′). We digested the PCR products with PdmI (XmnI) (Thermo Scientific). The diploid allele gives digestion products of ∼200 and ∼100 bp. The tetraploid allele remains undigested. To genotype for ASY3 D vs. T alleles, we amplified an ∼300-bp fragment of the ASY3 gene with primers F1 (5′-GCC​CTG​AAA​ATG​CTA​CCA​GAA​GGC​CAG​TGA-3′) and R1 (5′-GCG​AAA​ATG​AGC​CTT​ACG​AC-3′). We digested the PCR product with NmuCI (Tsp45I) restriction enzyme (Thermo Scientific). The diploid allele gives digestion products of ∼200 and ∼100 bp while the tetraploid allele remains undigested.

### Metaphase I Spreads.

For metaphase spreads, we followed the protocol described in ref. 55 with minor modifications. Briefly, we fixed inflorescences in 3:1 ethanol:acetic acid. Anthers were isolated and subsequently incubated in 300 μL of enzyme mixture (0.3% cellulase, 0.3% pectolyase in 10 mM citrate buffer) in a moist chamber at 37 °C for 90 min. Two buds were transferred to ∼2 μL of 80% acetic acid on a slide and macerated with a brass rod. Ten microliters of 80% acetic acid was then added to the slide, and the slide was placed on a hot block for 30 s before adding another 10 μL of 80% acetic acid and leaving the slide on the hot block for another 30 s; 2 × 200 μL of 3:1 fixative was then added to the slide before drying the back of the slide with a hairdryer. Slides were then mounted in 7 μL 1 μg/mL DAPI in Vectashield mounting medium (Vector Laboratories). All images are freely available in ref. 56.

### Immunocytology.

For immunostaining of *A. arenosa* pachytene cells, we followed the protocol described in ref. 54 with minor modifications. Briefly, anthers containing meiocytes of the desired meiotic stage were dissected from fresh buds and macerated on a No.1.5H coverslip (Marienfeld) in 10 µL digestion medium (0.4% cytohelicase [Sigma], 1.5% sucrose, 1% polyvinylpyrrolidone [Sigma] in sterile water) for 1 min using a brass rod. Coverslips were then incubated in a moist chamber at 37 °C for 4 min before adding 10 µL of 2% Lipsol solution (SciLabware) followed by 20 µL 4% paraformaldehyde (pH 8). Once coverslips were dry, they were blocked in 0.3% bovine serum albumin in phosphate-buffered saline (PBS) and then incubated with primary antibody overnight at 4 °C and secondary antibody for 2 h at 37 °C. Before and after each antibody incubation coverslips were washed in 1 × PBS solution plus 0.1% Triton X-100 (Sigma). Coverslips were finally incubated in 10 µg/mL DAPI for 5 min before being mounted on a slide in 7 µL Vectashield (Vector Laboratories). The following primary antibodies were used at 1:500 dilutions: anti-ASY1 (rat and guinea pig), anti-ZYP1 (rat and guinea pig), and anti-HEI10 (rabbit). The following secondary antibodies were used at 1:200 dilutions: anti-rat Alexa Fluor 488 (Thermo Fisher), anti-rat Alexa Fluor 555 [F(ab′)2 fragment, Abcam], anti-guinea pig Alexa Fluor 488 (Thermo Fisher), and anti-rabbit Alexa Fluor 647 [F(ab')2 fragment, Thermo Fisher]. Immunostained cells were imaged using four-color structured illumination microscopy (3D-SIM) on a Zeiss Elyra PS1 microscope. All SC length measurements were performed in three dimensions, and 3D models of each cell were generated using using the Simple Neurite Tracer ImageJ Plugin (57). All images are freely available in ref. 56.

### Statistical Analysis.

Statistical analyses were performed using R. For metaphase I data analysis, the chisq.test() and pairwiseNominalIndependence() functions from the stats and rcompanion packages, respectively, were used for χ^2^ testing with post hoc pairwise analysis on data pooled from biological replicates (plants) of each genotype. For the ASY1 experiment only, counts from diploid cells were doubled to enable comparison with tetraploid cells. As pooling data from biological replicates left this analysis vulnerable to type I error due to possible sacrificial pseudoreplication, we performed follow-up Poisson-GLMM and Binomial-GLMM analysis using “genotype” as a fixed factor and “plant” as a random factor. We obtained estimated means, SEs, and between-genotype *P* values for each genotype by refitting GLMM models using each genotype as a reference factor. Normal approximations of 95% confidence intervals were calculated as 1.96 estimated SEs (*SI Appendix*, Table S3). Poisson-GLMMs were fitted using the glmer() function of the lme4 R package (58). Poisson distributions were confirmed by checking that the variance within the data were approximately equal to the mean. Estimated means, SEs, and between-genotype *P* values for each genotype in each experiment were obtained by refitting GLMM models using each genotype as a reference factor. Immunocytological measurements were analyzed using a similar mixed-effects model/nested ANOVA approach by utilizing the lmer() function in R. For nested ANOVAs, post hoc Tukey comparisons of means were performed using the glht() function. Normal approximations of 95% confidence intervals were calculated as 1.96 estimated SEs. Models were validated by plotting residuals vs. fitted values and residuals vs. each variable and checking for overdispersion. Kolmogrov–Smirnov tests were used to test for differences in the distribution of single HEI10 focus positions between genotypes. Example R scripts and accompanying.CSV files for GLMM analysis are provided in *SI Appendix*.

### Sequence Polymorphism.

Sequence polymorphisms in ASY1 and ASY3 were described in previous publications (24, 38). We reused published bam files from ref. 24 and called variants using GATK v.3.5, following GATK best practices. To confirm polymorphisms in D and T alleles, we sequenced cDNAs from meiocytes of TBG tetraploid plants (D and T alleles in tetraploids) and SN diploids (D alleles in the colchiploids) and for ASY3 from another tetraploid population (TRE) that has not had gene flow from diploids (23, 24). We isolated RNA from developing anthers frozen in liquid nitrogen. We extracted RNA using the RNAqueous-Micro total RNA isolation kit (Ambion, Austin, TX) according to the manufacturer’s instructions. Quality and quantity of RNA were determined using an Agilent 2100 Bioanalyzer (Agilent Technologies, Waldbronn, Germany). We synthesized cDNA from 5 μg of total RNA using the SuperScript III First-Strand Synthesis Kit (Invitrogen) following the manufacturer’s instructions. ASY1 cDNAs were amplified with the following primers: Forward—ATGGTGATGGCTCAGAAGCTGAAG; Reverse—ATTAGCTTGAGATTTCTGACGCTT. ASY3 cDNAs were amplified with the following primers: Forward—ATGAGCGACTATAGAAGTTTCGGC; Reverse—ATCATCCCTCAAACATTCTGCCAC. We cloned PCR products into pMiniT 2.0 vectors with the NEB PCR Cloning Kit (New England Biolabs) for dideoxy sequencing. For each individual, we sequenced three to five independent amplicons.

### Data Availability.

All cytological images on which our analyses are based are freely available at https://www.research-collection.ethz.ch/handle/20.500.11850/386103 (DOI: 10.3929/ethz-b-000386103).

## Supplementary Material

Supplementary File

Supplementary File

Supplementary File
