# Supplementary material for: Derived alleles of two axis proteins affect meiotic traits in autotetraploid Arabidopsis arenosa
Source: Proc Natl Acad Sci U S A. 2020 Apr 9;117(16):8980–8. doi: 10.1073/pnas.1919459117 (PMC7183234; doi:10.1073/pnas.1919459117)
Supplement: Supplementary File [file pnas.1919459117.sapp.pdf]

PNAS
www.pnas.org

Supplementary Information for

Derived alleles of two axis proteins affect meiotic traits in autotetraploid *Arabidopsis arenosa*

Chris Morgan[1, §], Huakun Zhang[1,2, §], Clare E. Henry[1], F. Chris H. Franklin[3], Kirsten Bomblies*[,1,4]

1. John Innes Centre, Department of Cell and Developmental Biology, Colney Lane, Norwich, NR4 7UH, UK.
2. Key Laboratory of Molecular Epigenetics of Ministry of Education, Northeast Normal University, Changchun 130024, China
3. School of Biosciences, The University of Birmingham, Edgbaston, Birmingham, B15 2TT, UK.
4. Institute of Molecular Plant Biology, Department of Biology, ETH, Universitätsstrasse 2, 8092 Zürich, Switzerland.

§ These authors contributed equally.

Author for correspondence: Email: Kirsten.bomblies@biol.ethz.ch

This PDF file includes:

Figures S1 to S5
Tables S1 to S8
SI References

Separate files are provided that contain .csv files with sample datasets and sample R scripts for GLMM.

Figure S1: Alignments of ASY1 D and T alleles.

```
ASY1_2XSN_Cons   MVMAQKLKEAEITEQDSLLLTRNLLRIAIFNISYIRGLFPEKYFNDKSVPALDMKIKKLM   60
ASY1_D           MVMAQKLKEAEITEQDSLLLTRNLLRIAIFNISYIRGLFPEKYFNDKSVPALDMKIKKLM   60
ASY1_4XTBG_Cons  MVMAQKLKEAEITEQDSLLLTRNLLRIAIFNISYIRGLFPEEYFNDKSVPALDMKIKKLM   60
ASY1_T-1         MVMAQKLKEAEITEQDSLLLTRNLLRIAIFNISYIRGLFPEEYFNDKSVPALDMKIKKLM   60
ASY1_T-2         MVMAQKLKEAEITEQDSLLLTRNLLRIAIFNISYIRGLFPEEYFNDKSVPALDMKIKKLM   60

ASY1_2XSN_Cons   PMDAESRRLIDWMEKGVYDALQRKYLKTLMFCICETVDGPMIEEYAFSFSYSDSDSQDVM  120
ASY1_D           PMDAESRRLIDWMEKGVYDALQRKYLKTLMFCICETVDGPMIEEYAFSFSYSDSDSQDVM  120
ASY1_4XTBG_Cons  PMDAESRRLIDWMEKGVYDALQRKYLKTLMFCICETVDGPMIEEYAFSFSYSDSDSQDVM  120
ASY1_T-1         PMDAESRRLIDWMEKGVYDALQRKYLKTLMFCICETVDGPMIEEYAFSFSYSDSDSQDVM  120
ASY1_T-2         PMDAESRRLIDWMEKGVYDALQRKYLKTLMFCICETVDGPMIEEYAFSFSYSDSDSQDVM  120

ASY1_2XSN_Cons   MNINRTGNKKNGGTFNSTADITPNQMRSSACKMVRTLVQLMRTLDKMPDERTIVMKLLYY  180
ASY1_D           MNINRTGNKKNGGTFNSTADITPNQMRSSACKMVRTLVQLMRTLDKMPDERTIVMKLLYY  180
ASY1_4XTBG_Cons  MNINRTGNKKNGGTFNSTADITPNQMRSSACKMVRTLVQLMRTLDKMPDERTIVMKLLYY  180
ASY1_T-1         MNINRTGNKKNGGTFNSTADITPNQMRSSACKMVRTLVQLMRTLDKMPDERTIVMKLLYY  180
ASY1_T-2         MNINRTGNKKNGGTFNSTADITPNQMRSSACKMVRTLVQLMRTLDKMPDERTIVMKLLYY  180

ASY1_2XSN_Cons   DDVTPPDYEPPFFRGCTEDEAQYVWTKNPLRMEIGNVNSKHLVLTLKVKSVLDPCEDEND  240
ASY1_D           DDVTPPDYEPPFFRGCTEDEAQYVWTKNPLRMEIGNVNSKHLVLTLKVKSVLDPCEDEND  240
ASY1_4XTBG_Cons  DDVTPPDYEPPFFRGCTEDEAQYVWTKNPLRMEIGNVNSKHLVLTLKVKSVLDPCEDEND  240
ASY1_T-1         DDVTPPDYEPPFFRGCTEDEAQYVWTKNPLRMEIGNVNSKHLVLTLKVKSVLDPCEDEND  240
ASY1_T-2         DDVTPPDYEPPFFRGCTEDEAQYVWTKNPLRMEIGNVNSKHLVLTLKVKSVLDPCEDEND  240

ASY1_2XSN_Cons   DMQDDGKSIGPDSVHDDQPSDSDSEISQTQENQFIVAPVEKQDDDDGEVDEGDNTQDPVE  300
ASY1_D           DMQDDGKSIGPDSVHDDQPSDSDSEISQTQENQFIVAPVEKQDDDDGEVDEDDNTQDPVE  300
ASY1_4XTBG_Cons  DMQDDGKSIGPDSVHDDQPSDSDSEISQTQENQSIVAPVEKQDDDDGEVDEGDNTQDPVE  300
ASY1_T-1         DMQDDGKSIGPDSVHDDQPSDSDSEISQTQENQSIVAPVEKQDDDDGEVDEDDNTQDPVE  300
ASY1_T-2         DMQDDGKSIGPDSVHDDQPSDSDSEISQTQENQSIVAPVEKQDDDDGEVDEDDNTQDPVE  300

ASY1_2XSN_Cons   NEQQLARVKDWINSRHLDTLELTDILANFPDISIVLSEEIMDQLVTEGVLSKTGKDTYIK  360
ASY1_D           NEQQLARVKDWINSRHLDTLELTDILANFPDISIVLSEEIMDQLVTEGVLSKTGKDTYIK  360
ASY1_4XTBG_Cons  NEQQLARVKDWINSHHLDTLELTDILANFPDISIVLSEEIMDQLVTEGVLSKTGKDTYIK  360
ASY1_T-1         NEQQLARVKDWINSHHLDTLELTDILANFPDISIVLSEEIMDQLVTEGVLSKTGKDTYIK  360
ASY1_T-2         NEQQLARVKDWINSHHLDTLELTDILANFPDISIVLSEEIMDQLVTEGVLSKTGKDTYIK  360

ASY1_2XSN_Cons   KRDKQTPESEFTFVKEEADGQTAPKDGKPVAPEDYLYMKALYHSLPMNYVTITKLHNMLD  420
ASY1_D           KRDK-TPECEFTFVKEEADGQTALKDGKPVAPEDYLYMKALYHSLPMNYVTITKLHNMLD  419
ASY1_4XTBG_Cons  KRDKQTPESEFTFVKEEADGQTAPKDGKPVAPEDYLYMKALYHSLPMKYVTITKLHNMLD  420
ASY1_T-1         KRDK-TPESEFTFVKEEADGQTAPKDGKPVAPEDYLYMKALYHSLPMKYVTITKLHNMLD  419
ASY1_T-2         KRDKQTPESEFTFVKEEADGQTAPKDGKPVAPEDYLYMKALYHSLPMKYVTITKLHNMLD  420
                     ^

ASY1_2XSN_Cons   GEANQTAVRKLMDRMTQEGYVEASSNRRLGKRVIHSSLTERKLNEVRKVLDTDDMDVDVN  480
ASY1_D           GEANQTAVRKLMDRMTQEGYVEASSNRRLGKRVIHSSLTERKLNEVRKVLDPDDMDVDVN  479
ASY1_4XTBG_Cons  GEANQTAVRKLMDRMTQEGYVEASSNRRLGKRVIHSSLTERKLNEVRKVLDTDDMDVDVN  480
ASY1_T-1         GEANQTAVRKLMDRMTQEGYVEASSNRRLGKRVIHSSLTERKLNEVRKVLDTDDMDVDVN  479
ASY1_T-2         GEANQTAVRKLMDRMTQEGYVEASSNRRLGKRVIHSSLTERKLNEVRKVLDTDDMDVDVN  480

ASY1_2XSN_Cons   EAINKTNGLEAKVTADVSTCGGIHSIGSDFTRTKGRSGGMQQNGSVLSEQTISKAGNTPI  540
ASY1_D           EAINKTNGLEAKVTADVSTCGGIHSIGSDFTRTKGRSGGMQQNGSVLSEQTISKAGNTPI  539
ASY1_4XTBG_Cons  EAINKTNGLEAKVTADVSTCGGIHSIGSDFTRTKGRSGGMQQNGSVLSEQTISKAGNTPI  540
ASY1_T-1         EAINKTNGLEAKVTADVSTCGGIHSIGSDFTRTKGRSGGMQQNGSVLSEQTISKAGNTPI  539
ASY1_T-2         EAINKTNGLEAKVTADVSTCGGIHSIGSDFTRTKGRSGGMQQNGSVLSEQTISKAGNTPI  540

ASY1_2XSN_Cons   SNKAQPAASRESFAVNGGAAKEVETVNCSQASQDRRGRKTSMVRDPILQYSKRQKSQAN   599
ASY1_D           SNKAQPAASRESFAVHGATAKEAETVNCSQASQDRRGRKTSMVRDPILQYSKRQKSQAN   598
ASY1_4XTBG_Cons  SNKAQPAASRESFAVHGATAKEAETVNCSRASQDRRGRKTSMVRDPILQYSKRQKSQAN   599
ASY1_T-1         SNKAQPAASRESFAVHGATAKEAETVNCSRASQDRRGRKTSMVRDPILQYSKRQKSQAN   598
ASY1_T-2         SNKAQPAASRESFAVHGATAKEAETVNCSRASQDRRGRKTSMVRDPILQYSKRQKSQAN   599
```

Figure S1. Alignment of ASY1 protein sequences. ASY1_2XSN_Cons and ASY1_4XTBG_Cons are consensus sequences from short read sequencing published in Monnahan et al 2019 (1) from the diploid SN population from which our colchiploids were generated, and TBG, the tetraploid from which all of the T alleles, and the D alleles used in the ASY1 experiment were derived. ASY1_D and ASY1_T-1 and ASY1_T-2 are D and T allele cDNAs. Polymorphisms marked in black that differentiate D and T alleles are identical to those previously reported and described in detail in Wright et al 2015 (2). There are three additional polymorphisms in the D allele that were not previously reported (highlighted in blue). The D allele carries three additional amino acid changes not previously detected (highlighted in blue), and the consensus sequence has four calls not represented in the cDNAs (red). Arrowhead indicates a presence/absence polymorphism of Q365 that arises from alternate splicing; this occurs in both diploids and tetraploids. A yellow bar below the alignment marks the conserved HORMA domain and green marks a SWIRM domain. The red boxes in the SN consensus sequence represent sites where the consensus calculated from short read data deviates from all sequences we obtained from cDNA in both ploidies.

Figure S2: Alignments of ASY3 D and T alleles.

```
ASY3_4X_TBG_CONS  MSDYRSFGSNYHPSSHSRKISIGVMADSQPKRNLVPDKDDGDVIARVEKLKSAPVTDLQA   60
DDTT4_T_ALLELE    MSDYRSFGSNYHPSSHSRKISIGVMADSQPKRNLVPDKDDGDVIARVEKLKSAPVTDLQA   60
TRE1_T            MSDYRSFGSNYHPSSHSRKISIGVMADSQPKRNLVPDKDDGDVIARVEKLKSAPVTDLQA   60
DDTT1_D_ALLELE    MSDYRSFGSNYHPSSQSRKISIGVMADSQPKRNLVPNKDDGDVIARAEKLKSATVTDLQV   60
ASY3_2X_SN_CONS   MSDYRSFGSNYHPSSQSRKISIGVMADSQPKRNLVPNKDDGDVLARVEKLKSATVTDLQA   60

ASY3_4X_TBG_CONS  NKKDKGDLAAKQRNSAQVTGHVTSPWRSPRSSHRKSGTLESVLCKQTSSLSGSKGLTKGL  120
DDTT4_T_ALLELE    NKKDKGDLAAKQRNSAQVTGHVTSPWRSPRSSHRKSGTLESVLCKQTSSLSGSKGLTKGL  120
TRE1_T            NKKDKGDLAAKQRNSAQVTGHVTSPWRSPRSSHRKSGTLESVLCKQTSSLSGSKGLTKGL  120
DDTT1_D_ALLELE    KKKDKGDLAAKQRNSAQVTGHVTSPWRSPRSSHRKLGTLENVLCKQTSSLSGSKGLNKGI  120
ASY3_2X_SN_CONS   NKKDKGDLAAKQRNSAQVTGHVTTPWRSPRSSHRKLGTLESVLCKQTSSLSGSKGLNKGL  120

ASY3_4X_TBG_CONS  NGAHPAPARDSFPNFPISSPQQSHGELNGGRNDTVMDRSPERMEDPPSAVLLQKVASQRE  180
DDTT4_T_ALLELE    NGAHPAPARDSFPNFPISSPQQSHGELNGGRNDTVMDRSPERMEDPPSAVLLQKVASQRE  180
TRE1_T            NGAHPAPARDSFPNFPISSPQQSHGELNGGRNDTVMDRSPERMEDPPSAVLLQKVASQRE  180
DDTT1_D_ALLELE    NGAHPVPARDSFQNFPISSPQHSHGELNGGRSNTVMDRSPERMEDPPSAVLQQKVASQRE  180
ASY3_2X_SN_CONS   NGAHPAPARDSFQNFPISSPQQSHGELNGGRNDTVMDRISERMEDPPSAVLQQKVASQRE  180

ASY3_4X_TBG_CONS  MDKPDKEKNGTTVVLRSKLWEILGKASTANNEDVNSATPEVEKTNFKLSQDKGSNDDPLI  240
DDTT4_T_ALLELE    MDKPDKEKNGTTVVLRSKLWEILGKASTANNEDVNSATPEVEKTNFKLSQDKGSNDDPLI  240
TRE1_T            MDKPDKEKNGTTVVLRSKLWEILGKASTANNEDVNSATPEVEKTNFKLSQDKGSNDDPLI  240
DDTT1_D_ALLELE    MDKPDKEKNGTTDVLRSKLWEILGKASPANNEDVNSETPEVEKTNFRLSQDKGSNDDPVI  240
ASY3_2X_SN_CONS   MDKPDKEKNGTTDVLRSKLWEILGKASPANNEDVNSETPEVEKTNFKLSQDKGSNDDPLI  240

ASY3_4X_TBG_CONS  KPRHNSDSIETDSESPENATRRPVIRSVLQRRVGTKGVQKRTKAGANLGRKSTEQVNDVF  300
DDTT4_T_ALLELE    KPRHNSDSIETDSESPENATRRPVIRSVLQRRVGTKGVQKRTKAGANLGRKSTEQVNDVF  300
TRE1_T            KPRHNSDSIETDSESPENATRRPVIRSVLQRRVGTKGVQKRTKAGANLGRKSTEQVNDVF  300
DDTT1_D_ALLELE    KPRHNSDSIETDSESPEIATRRPVTRSLLQRKVGAKGVQKRTKAGANLGRKSTEQVNNVF  300
ASY3_2X_SN_CONS   KPRHNSDSIETDSESPENATRRPVTRSLLQRRVGAKGVQKRTKAGANLGRKSTEQVNNVF  300

ASY3_4X_TBG_CONS  SFEEGLRGKIGTAMNSSVIPKKQRGRRKNTVVQCRKAHFRRKDEADGSRKEASKNNTPPR  360
DDTT4_T_ALLELE    SFEEGLRGKIGTAMNSSVIPKKQRGRRKNTVVQCRKAHFRRKDEADGSRKEASKNNTPPR  360
TRE1_T            SFEEGLRGKIGTAMNSSVIPKKQRGRRKNTVVQCRKAHFRRKDEADGSRKEASKNNTPPR  360
DDTT1_D_ALLELE    SFEEGLRGKIGTAMNSSVMPKKQRGRRKNTVVQCRKAHFRRNDEADGSRKETSKSNTPPR  360
ASY3_2X_SN_CONS   SFEEGLRGKIGTAMNSSVIPKKQRARRKNTVVQCRKAHFRRNDEADGSHKEASKSNTPPR  360

ASY3_4X_TBG_CONS  SESTGTGKRSSFSDKKGSSQDLHPQSKARKQKPDISTREGDFHPSPEAETAAPEMFQGL  420
DDTT4_T_ALLELE    SESTGTGKRSSFSDKKGSSQDLHPQSKARKQKPDISTREGDFHPSPEAETAAPEMFQGL  420
TRE1_T            SESTGTGKRSSFSDKKGSSQDLHPQSKARKQKPDISTREGDFHPSPEAETAAPEMFQGL  420
DDTT1_D_ALLELE    SESTGTGKRSSFSDKKGSSHDLHPQSKARKQKPDISTREGDFHPSPEAETAAPEMFQGL  420
ASY3_2X_SN_CONS   SESTGTGKRSSFSDKKESSHDLHPQSKAQKKKPDISTREGDFHPSPEAETAAPEMFQGL  420

ASY3_4X_TBG_CONS  SKNGEKHERPSNIFMEKSVEPENEFQSPTFGYKAPISSPSPCFSPEASPLQPRNISPTLE  480
DDTT4_T_ALLELE    SKNGEKHERPSNIFMEKSVEPENEFQSPTFGYKAPISSPSPCFSPEASPLQPRNISPTLE  480
TRE1_T            SKNGKKHERPSNIFMEKSVEPENEFQSPTFGYKAPISSPSPCFSPEASPLQPRNISPTLE  480
DDTT1_D_ALLELE    SKNGEKHERPSNVFMEKSIEPENEFQSPTFGYKAPISSPSPCLSPEASPLQPPNISPTLE  480
ASY3_2X_SN_CONS   SKNGEKHERPSNVFMEKSVEPENEFQSPTFGYKAPISSPSPCFSPEVSPLQPRNISPTLE  480

ASY3_4X_TBG_CONS  EMETPIFSFGTKKNSQGTTGQASDTERRLPEFLEKK------------------------  516
DDTT4_T_ALLELE    EMETPIFSFGTKKNSQGTTGQASDTERRLPEFLEKKRDYSFRRESSPEPKEDLVLSDSSS  540
TRE1_T            EMETPIFSFGTKKNSQGTTAQASDTERRLPEFLEKKRDYSFRRESSPEPKEDLVLSDSSS  540
DDTT1_D_ALLELE    ETETPIFSFGTKKTSQGTTGQASDTERRLPDFLEKK------------------------  516
ASY3_2X_SN_CONS   ETETPIFSFGTKKTSQGTTGQASDTERRLPDFLEKK------------------------  516
                                                            XXXXXXXXXXXXXXX
                                              (X=predicted SUMOylation site, T only)

ASY3_4X_TBG_CONS  --RDYSFRRESSPEPKEDLVLSDSSSDERDSDGSGEDSPVLGHNNSPEERKTSNWSNEKS  574
DDTT4_T_ALLELE    DERDYSFRRESSPEPKEDLVLSDSSSDERDSDGSGEDSPVLGHNNSPEERKTSNWSNEKS  600
TRE1_T            DERDYSFRRESSPEPKEDLVLSDSSSDERDSDGSGEDSPVLGHNNSPEERKTSNWSNEKS  600
DDTT1_D_ALLELE    --RDYSFRRESSPEPNEDLVLSDPSSDERDSDGSGEDSPVLGHNNSPEERKTANWSNEKS  574
ASY3_2X_SN_CONS   --RDYSFRRESSPEPNEDLVLSDPSSDERDSDGSGEDSPVLSHNNSPEERKTANWSNEKS  574
                          XXXXXXXXXXXXXXX
                  (X=predicted SUMOylation site, T only)
```

```
ASY3_4X_TBG_CONS KLGSSSAKRNSNLKGIGRVVLSPPSSLSKGIDKTDSFQHCSEMDEDEDEGLGRAVALFAM  634
DDTT4_T_ALLELE   KLGSSSAKRNSNLKGIGRVVLSPPSSLSKGIDKTDSFQHCSEMDEDEDEGLGRAVALFAM  660
TRE1_T           KLGSSSAKRNSNLKGIGRVVLSPPSSLSKGIDKTDSFQHCSEMDEDEDEGLGRAVALFAM  660
DDTT1_D_ALLELE   KLGSSSAKRNSNLKGIGRVVLSPPSSLSKGIDKTDSFQHCSEMDEDEDEGLGRAVALFAM  634
ASY3_2X_SN_CONS  KLGSSSAKRNSNLKGIGRVVLSPPSSLSKGIDKTDSFQHCSEMDEDEDEGLGRAVALFAM  634

ASY3_4X_TBG_CONS ALQNFEKKLKSAAEKKSSEIIASVSEEIHLELENVKSHIITEAGKTSNLAKTKRKHAETR  694
DDTT4_T_ALLELE   ALQNFEKKLKSAAEKKSSEIIASVSEEIHLELENVKSHIITEAGKTSNLAKTKRKHAETR  720
TRE1_T           ALQNFEKKLKSAAERKSSEIIASVSEEIHLELENVKSHIITEAGKTSNLAKTKRKHAETR  720
DDTT1_D_ALLELE   ALQNFEKKLKSAAEKKSSEIIASVSEEIHLELENVKSHIITEAGKTSNLAKAKRKHAETR  694
ASY3_2X_SN_CONS  ALQNFEKKLKSAAEKKSSEIIASVSEEIHLELENVKSHIITEAGKTSNLAKTKRKHAETR  694

ASY3_4X_TBG_CONS LQEQQEKMRMIHEKFKDDVSHHLEDFKSTIEELEGNHSELKGSIKKQRTSHQKLIAHFEG  754
DDTT4_T_ALLELE   LQEQQEKMRMIHEKFKDDVSHHLEDFKSTIEELEGNHSELKGSIKKQRTSHQKLIAHFEG  780
TRE1_T           LQEQQEKMRMIHEKFKDDVSHHLEDFKSTIEELEGNHSELKGSIKKQRTSHQKLIAHFEG  780
DDTT1_D_ALLELE   LQEQQEKMRMIHEKFKDDVSHHLEDFKSTIEELEGNHSELKGSIKKQRTSHQKLIAHFEG  754
ASY3_2X_SN_CONS  LQEQQEKMRMIHEKFKDDVSHHLEDFKSTIEELEGNHSELKGSIKKQRTSHQKLIAHFEG  754
                         XXXXXXXXXXXXXXX

ASY3_4X_TBG_CONS GIETKLDNATKRIDSVNKSARGKMLQLKMIVAECLRDD                        792
DDTT4_T_ALLELE   GIETKLDNATKRIDSVNKSARGKMLQLKMIVAECLRDD                        818
TRE1_T           GIETKLDNATKRIDSVNKSARGKMLQLKMIVAECLRDD                        818
DDTT1_D_ALLELE   GIETKLDNATKRIDSVNKSARGKMLQLKMIVAECLRDD                        792
ASY3_2X_SN_CONS  GIETKLDNATKRIDSVNKSARGKMLQLKMIVAECLRDD                        792
```

Figure S2. Alignment of ASY3 protein sequences. ASY3_2X_SN_Cons and ASY1_4X_TBG_Cons are consensus sequences from short read sequencing published in (1) from the diploid SN population from which our colchiploids were generated, and TBG, the tetraploid from which all of the tetraploid alleles were derived. DDTT1 and DDTT4 are two cDNA sequences from a heterozygous tetraploid showing the D vs T alleles. All previously identified amino acid differences between the diploid and tetraploid alleles (2) could be confirmed from our sequencing (highlighted in black), and we found five additional amino acid changes (highlighted in red). The boxes highlighted in grey with white lettering (outside the duplication region) are amino acid differences between in any one sequence in the alignment, but not generally differentiated between ploidies.

The T allele has a duplication relative to the diploid sequence (highlighted in dark grey, duplicated sequence boxed), but also relative to the short-read consensus sequence for the tetraploids. To establish whether this duplication is characteristic of the tetraploids and its absence from the consensus an artefact of short read alignment, we sequenced cDNAs from an additional tetraploid population that lacks diploid alleles (TRE) and found that this allele also has the insertion suggesting that it is characteristic of the T allele. The duplication contains two polymorphisms in the adjacent region that are characteristic of the tetraploid, suggesting the duplication occurred in the context of an allele that already had these two amino acid differences.

Predicted SUMOylation consensus sites are marked with X's below the alignment. One of these is in the duplicated region and contains an amino acid difference between diploids and tetraploids such that in tetraploids there are two predicted SUMOylation sites and in the diploid allele, the site is not predicted to be SUMOylated (SUMOylation sites predicted by GPS-SUMO: http://sumosp.biocuckoo.org/online.php).

Figure S3: Scatterplots of HEI10 foci per cell and total SC length per cell (µm) in ASY1/ASY3 genotypes.

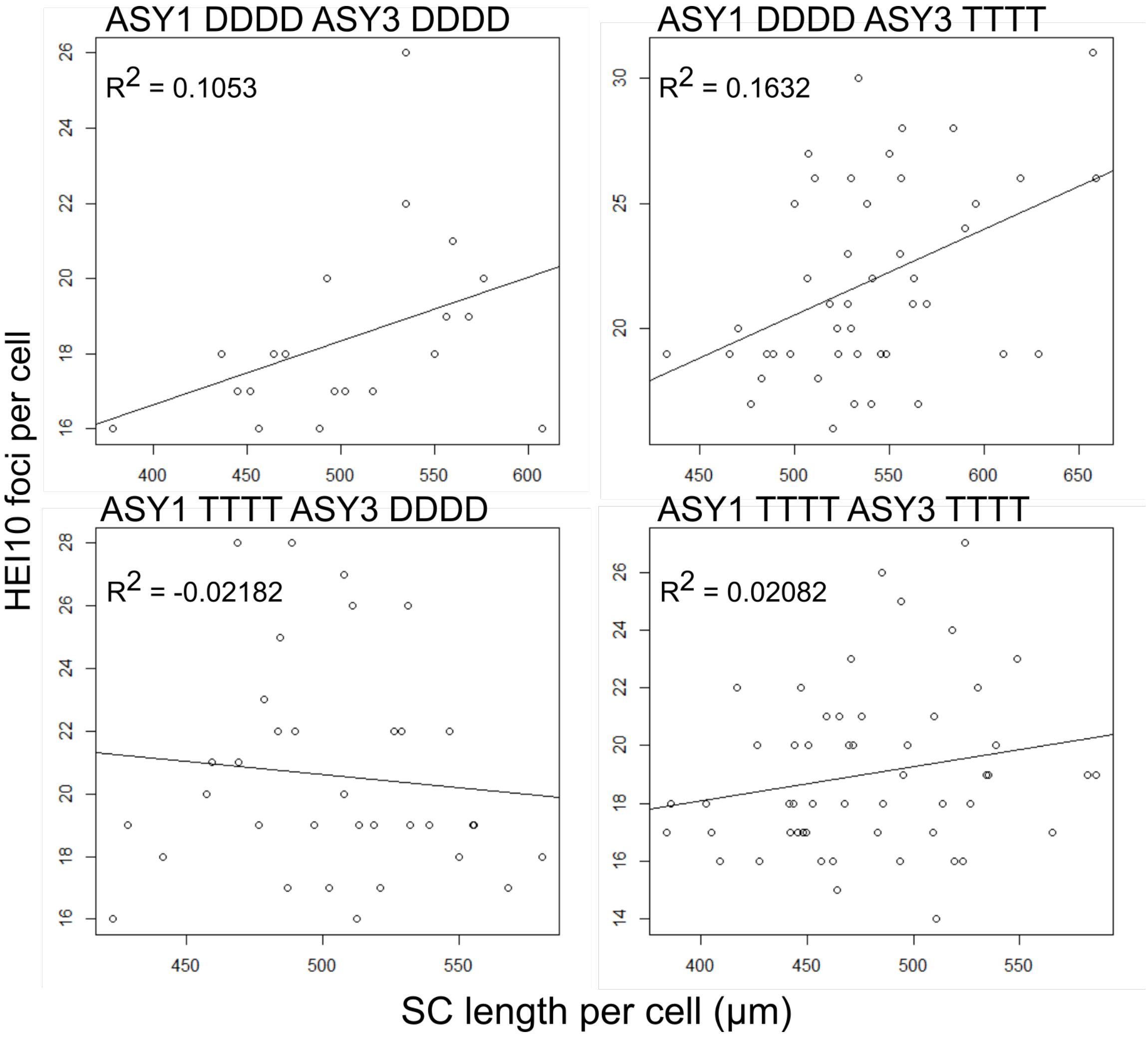

Figure S3: Scatterplots with fitted linear regression lines showing the relationship between the number of HEI10 foci per cell and total SC length per cell (µm) in different ASY1/ASY3 genotypes.

Figure S4: Using 4-colour SIM to determine CO positions along component chromosomes in tetraploid pachytene cells.

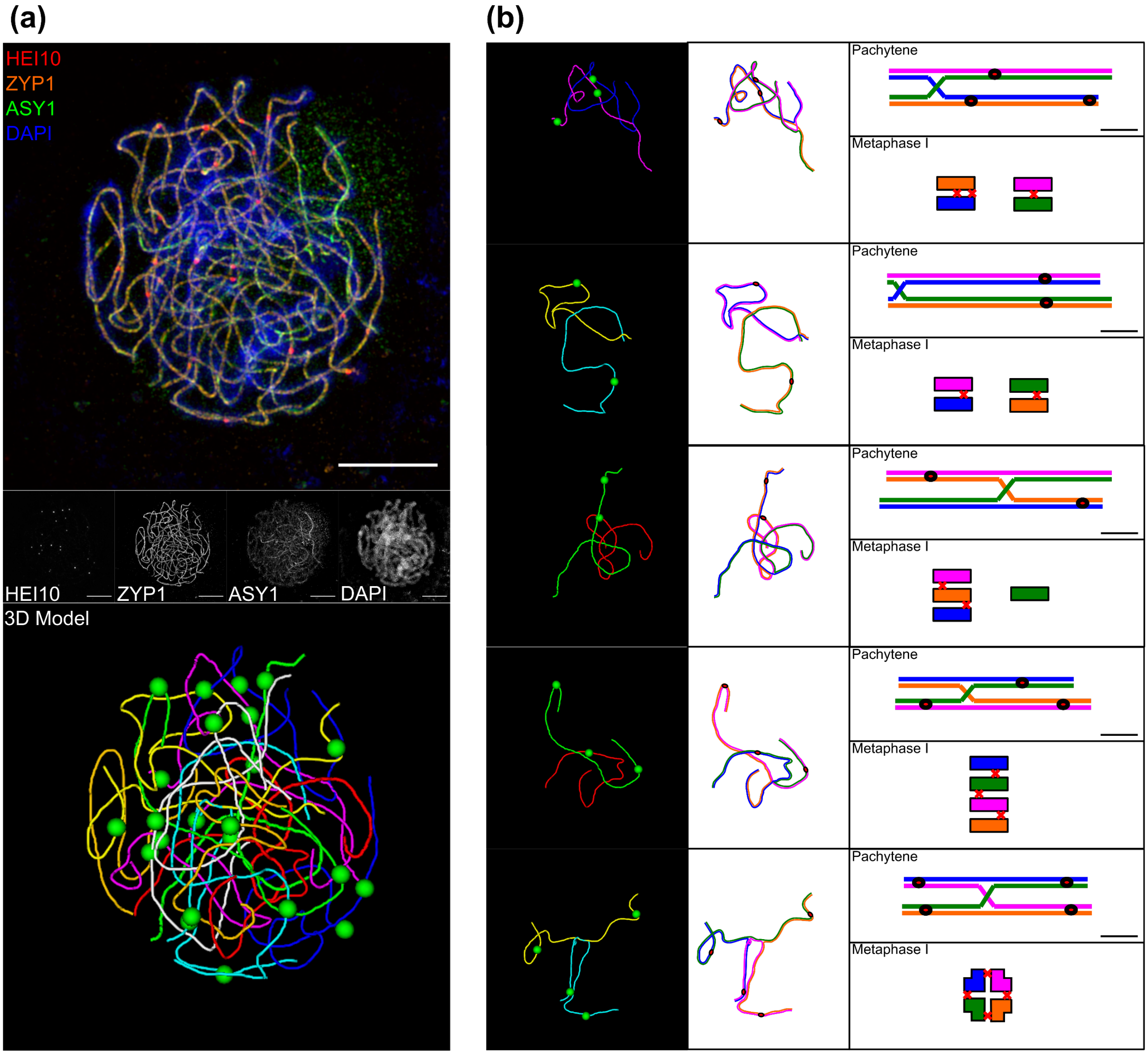

Figure S4: Using 4-colour SIM to determine CO positions along component chromosomes in tetraploid pachytene cells. (a) A tetraploid cell stained for HEI10, ASY1, ZYP1 and DAPI and imaged with 3D-SIM. A 3D rendering of the cell is shown in which different traced pairs of synapsed component chromosomes are labelled in different colours and green spheres show the positions of HEI10 foci. (b) This cell contains seven synaptic multivalents. The 3D traces of five synaptic multivalents are shown as well as a diagram illustrating the synaptic behaviour of each component chromosome (labelled in different colours) and the positions of HEI10 foci (red dots). A simplified diagram of the component chromosome interactions (scales bar 5 microns) and the expected metaphase I outcome following removal of the SC are also shown (red crosses = chiasma).

Figure S5: Crossover / SPS configurations predicted metaphase I outcomes.

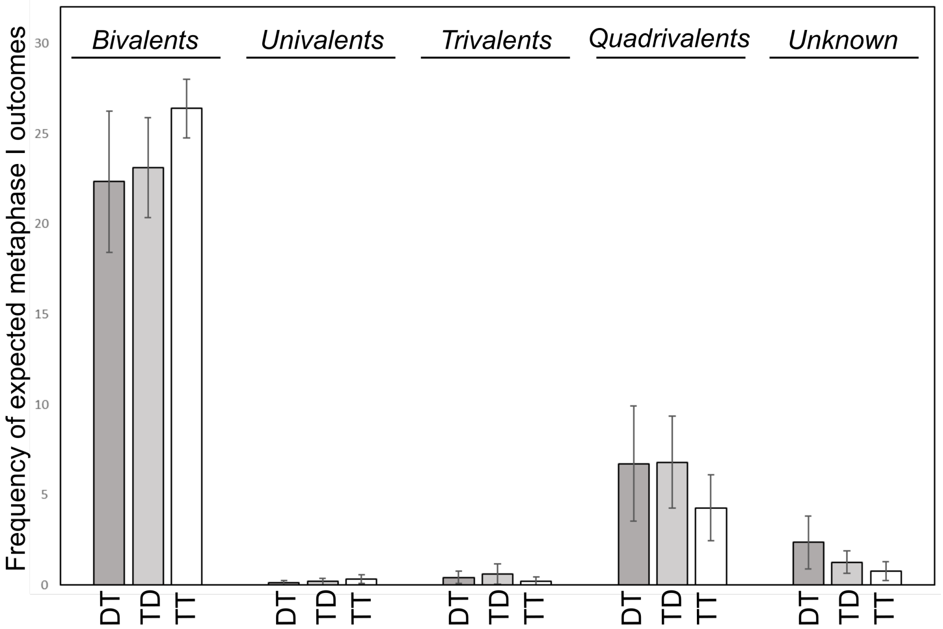

Figure S5. The frequency (in chromosomes per cell) of expected metaphase I outcomes based on pachytene late-HEI10/SPS site position in DT, TD and TT individuals. Unknown metaphase I outcomes arise from situations where HEI10 foci are separated by two or more SPS sites. Plots show the unweighted mean values from 4-5 individuals from each genotype ± sample standard deviation.

Table S1: Cytological trait values for *A. arenosa* tetraploids homozygous for diploid or tetraploid alleles of ASY1

								Proportions (out of total scorable bivalents)					
**Image**	**Genotype**	**Exp**	**O**	**+**	**ł**	**I**	**Ts**	**O**	**+**	**ł**	**I**	**EstCO/Bv**	**MV**
S-10	SN29 (Dip)	1	2	2	2	2	8	0.25	0.25	0.25	0.25	1.25	
S-104	SN29 (Dip)	1	3	2	2	1	8	0.38	0.25	0.25	0.13	1.38	
S_105	SN29 (Dip)	1	1	2	0	4	7	0.14	0.29	0.00	0.57	1.14	
S_106	SN29 (Dip)	1	3	1	2	1	7	0.43	0.14	0.29	0.14	1.43	
S_110	SN29 (Dip)	1	1	6	1	0	8	0.13	0.75	0.13	0.00	1.13	
S_112	SN29 (Dip)	1	1	3	1	1	6	0.17	0.50	0.17	0.17	1.17	
S_12	SN29 (Dip)	1	1	2	3	1	7	0.14	0.29	0.43	0.14	1.14	
S_127	SN29 (Dip)	1	4	1	3	0	8	0.50	0.13	0.38	0.00	1.50	
S_128	SN29 (Dip)	1	2	4	1	0	7	0.29	0.57	0.14	0.00	1.29	
S_131	SN29 (Dip)	1	2	1	1	1	5	0.40	0.20	0.20	0.20	1.40	
S_132	SN29 (Dip)	1	0	5	0	3	8	0.00	0.63	0.00	0.38	1.00	
S_134	SN29 (Dip)	1	0	3	2	2	7	0.00	0.43	0.29	0.29	1.00	
S_135	SN29 (Dip)	1	1	6	0	1	8	0.13	0.75	0.00	0.13	1.13	
S_144	SN29 (Dip)	1	3	2	1	1	7	0.43	0.29	0.14	0.14	1.43	
S_151	SN29 (Dip)	1	1	4	1	1	7	0.14	0.57	0.14	0.14	1.14	
S_154	SN29 (Dip)	1	0	2	5	1	8	0.00	0.25	0.63	0.13	1.00	
S_21	SN29 (Dip)	1	1	3	3	1	8	0.13	0.38	0.38	0.13	1.13	
S_3	SN29 (Dip)	1	2	4	1	1	8	0.25	0.50	0.13	0.13	1.25	
S_57	SN29 (Dip)	1	O	4	2	2	8	0.00	0.50	0.25	0.25	1.00	
S_59	SN29 (Dip)	1	1	2	1	1	5	0.20	0.40	0.20	0.20	1.20	
S_70	SN29 (Dip)	1	1	3	1	2	7	0.14	0.43	0.14	0.29	1.14	
S_72	SN29 (Dip)	1	0	3	2	3	8	0.00	0.38	0.25	0.38	1.00	
S_91	SN29 (Dip)	1	1	4	2	1	8	0.13	0.50	0.25	0.13	1.13	
S_92	SN29 (Dip)	1	1	5	1	1	8	0.13	0.63	0.13	0.13	1.13	
S_98	SN29 (Dip)	1	0	3	2	1	6	0.00	0.50	0.33	0.17	1.00	
21	TBG-2 TTTT	1	0	1	5	10	16	0.00	0.06	0.31	0.63	1.00	0
22	TBG-2 TTTT	1	1	3	4	3	11	0.09	0.27	0.36	0.27	1.09	1
36	TBG-2 TTTT	1	0	3	4	4	11	0.00	0.27	0.36	0.36	1.00	0
50	TBG-2 TTTT	1	1	5	2	1	9	0.11	0.56	0.22	0.11	1.11	1
58	TBG-2 TTTT	1	0	4	6	5	15	0.00	0.27	0.40	0.33	1.00	0
70	TBG-2 TTTT	1	0	5	7	3	15	0.00	0.33	0.47	0.20	1.00	1
82	TBG-2 TTTT	1	2	1	3	8	14	0.14	0.07	0.21	0.57	1.14	0
83	TBG-2 TTTT	1	0	7	6	3	16	0.00	0.44	0.38	0.19	1.00	0
86	TBG-2 TTTT	1	2	5	1	6	14	0.14	0.36	0.07	0.43	1.14	1
98	TBG-2 TTTT	1	1	2	4	6	13	0.08	0.15	0.31	0.46	1.08	0
100	TBG-2 TTTT	1	0	3	4	6	13	0.00	0.23	0.31	0.46	1.00	1
121	TBG-2 TTTT	1	2	0	1	13	16	0.13	0.00	0.06	0.81	1.13	1
138	TBG-2 TTTT	1	1	2	0	8	11	0.09	0.18	0.00	0.73	1.09	0
143	TBG-2 TTTT	1	3	4	5	4	16	0.19	0.25	0.31	0.25	1.19	0
155	TBG-2 TTTT	1	2	1	2	9	14	0.14	0.07	0.14	0.64	1.14	0

168	TBG-2 TTTT	1	0	4	6	5	15	0.00	0.27	0.40	0.33	1.00	1
184	TBG-2 TTTT	1	1	4	6	5	16	0.06	0.25	0.38	0.31	1.06	1
216	TBG-2 TTTT	1	3	0	3	3	9	0.33	0.00	0.33	0.33	1.33	1
251	TBG-2 TTTT	1	0	2	6	4	12	0.00	0.17	0.50	0.33	1.00	0
276	TBG-2 TTTT	1	2	2	5	7	16	0.13	0.13	0.31	0.44	1.13	0
277	TBG-2 TTTT	1	1	2	8	3	14	0.07	0.14	0.57	0.21	1.07	1
306	TBG-2 TTTT	1	2	0	5	6	13	0.15	0.00	0.38	0.46	1.15	0
310	TBG-2 TTTT	1	0	1	7	6	14	0.00	0.07	0.50	0.43	1.00	0
327	TBG-2 TTTT	1	0	0	2	6	8	0.00	0.00	0.25	0.75	1.00	1
329	TBG-2 TTTT	1	2	3	4	4	13	0.15	0.23	0.31	0.31	1.15	0
7	TBG-4 TTTT	1	3	1	9	2	15	0.20	0.07	0.60	0.13	1.20	0
8	TBG-4 TTTT	1	5	5	4	2	16	0.31	0.31	0.25	0.13	1.31	0
12	TBG-4 TTTT	1	3	7	3	2	15	0.20	0.47	0.20	0.13	1.20	0
19	TBG-4 TTTT	1	2	2	5	4	13	0.15	0.15	0.38	0.31	1.15	1
23	TBG-4 TTTT	1	2	5	6	3	16	0.13	0.31	0.38	0.19	1.13	0
75	TBG-4 TTTT	1	2	4	5	2	13	0.15	0.31	0.38	0.15	1.15	1
88	TBG-4 TTTT	1	1	3	5	3	12	0.08	0.25	0.42	0.25	1.08	0
108	TBG-4 TTTT	1	0	3	6	4	13	0.00	0.23	0.46	0.31	1.00	0
142	TBG-4 TTTT	1	3	3	3	7	16	0.19	0.19	0.19	0.44	1.19	0
176	TBG-4 TTTT	1	1	7	3	4	15	0.07	0.47	0.20	0.27	1.07	0
188	TBG-4 TTTT	1	0	5	6	2	13	0.00	0.38	0.46	0.15	1.00	1
191	TBG-4 TTTT	1	2	3	5	5	15	0.13	0.20	0.33	0.33	1.13	0
202	TBG-4 TTTT	1	0	6	5	5	16	0.00	0.38	0.31	0.31	1.00	0
203	TBG-4 TTTT	1	0	4	5	0	9	0.00	0.44	0.56	0.00	1.00	1
208	TBG-4 TTTT	1	3	1	5	1	10	0.30	0.10	0.50	0.10	1.30	1
211	TBG-4 TTTT	1	0	2	13	0	15	0.00	0.13	0.87	0.00	1.00	0
235	TBG-4 TTTT	1	1	5	0	3	9	0.11	0.56	0.00	0.33	1.11	1
248	TBG-4 TTTT	1	1	5	4	6	16	0.06	0.31	0.25	0.38	1.06	0
269	TBG-4 TTTT	1	0	3	10	1	14	0.00	0.21	0.71	0.07	1.00	0
305	TBG-4 TTTT	1	0	5	5	4	14	0.00	0.36	0.36	0.29	1.00	0
317	TBG-4 TTTT	1	1	3	7	1	12	0.08	0.25	0.58	0.08	1.08	0
323	TBG-4 TTTT	1	2	3	8	3	16	0.13	0.19	0.50	0.19	1.13	0
332	TBG-4 TTTT	1	4	4	3	2	13	0.31	0.31	0.23	0.15	1.31	0
333	TBG-4 TTTT	1	2	3	3	7	15	0.13	0.20	0.20	0.47	1.13	0
344	TBG-4 TTTT	1	3	6	6	1	16	0.19	0.38	0.38	0.06	1.19	0
14	TBG6A.1 7x8-3 TTTT	2	2	4	6	4	16	0.13	0.25	0.38	0.25	1.13	1
42	TBG6A.1 7x8-3 TTTT	2	2	0	2	5	9	0.22	0.00	0.22	0.56	1.22	1
72	TBG6A.1 7x8-3 TTTT	2	1	3	3	5	12	0.08	0.25	0.25	0.42	1.08	0
89	TBG6A.1 7x8-3 TTTT	2	0	2	3	11	16	0.00	0.13	0.19	0.69	1.00	0
97	TBG6A.1 7x8-3 TTTT	2	4	3	2	7	16	0.25	0.19	0.13	0.44	1.25	0
104	TBG6A.1 7x8-3 TTTT	2	5	2	3	4	14	0.36	0.14	0.21	0.29	1.36	1
123	TBG6A.1 7x8-3 TTTT	2	4	2	4	3	13	0.31	0.15	0.31	0.23	1.31	1
147	TBG6A.1 7x8-3 TTTT	2	2	3	5	4	14	0.14	0.21	0.36	0.29	1.14	0

154	TBG6A.1 7x8-3 TTTT	2	2	2	5	6	15	0.13	0.13	0.33	0.40	1.13			0
162	TBG6A.1 7x8-3 TTTT	2	3	5	4	4	16	0.19	0.31	0.25	0.25	1.19			0
163	TBG6A.1 7x8-3 TTTT	2	0	1	6	7	14	0.00	0.07	0.43	0.50	1.00			1
181	TBG6A.1 7x8-3 TTTT	2	2	0	2	11	15	0.13	0.00	0.13	0.73	1.13			0
184	TBG6A.1 7x8-3 TTTT	2	4	1	5	5	15	0.27	0.07	0.33	0.33	1.27			0
187	TBG6A.1 7x8-3 TTTT	2	1	4	4	3	12	0.08	0.33	0.33	0.25	1.08			0
194	TBG6A.1 7x8-3 TTTT	2	0	6	8	1	15	0.00	0.40	0.53	0.07	1.00			0
196	TBG6A.1 7x8-3 TTTT	2	3	3	5	5	16	0.19	0.19	0.31	0.31	1.19			0
197	TBG6A.1 7x8-3 TTTT	2	3	1	4	4	12	0.25	0.08	0.33	0.33	1.25			0
200	TBG6A.1 7x8-3 TTTT	2	4	2	2	6	14	0.29	0.14	0.14	0.43	1.29			0
203	TBG6A.1 7x8-3 TTTT	2	0	3	4	4	11	0.00	0.27	0.36	0.36	1.00			1
214	TBG6A.1 7x8-3 TTTT	2	3	5	4	3	15	0.20	0.33	0.27	0.20	1.20			0
226	TBG6A.1 7x8-3 TTTT	2	3	2	7	2	14	0.21	0.14	0.50	0.14	1.21			0
228	TBG6A.1 7x8-3 TTTT	2	0	0	11	3	14	0.00	0.00	0.79	0.21	1.00			0
262	TBG6A.1 7x8-3 TTTT	2	2	3	5	2	12	0.17	0.25	0.42	0.17	1.17			0
294	TBG6A.1 7x8-3 TTTT	2	2	1	5	4	12	0.17	0.08	0.42	0.33	1.17			0
295	TBG6A.1 7x8-3 TTTT	2	1	4	6	2	13	0.08	0.31	0.46	0.15	1.08			0
3	TBG6A.1 7x8-7 TTTT	2	4	2	0	7	13	0.31	0.15	0.00	0.54	1.31			0
8	TBG6A.1 7x8-7 TTTT	2	1	2	4	6	13	0.08	0.15	0.31	0.46	1.08			1
10	TBG6A.1 7x8-7 TTTT	2	1	3	4	5	13	0.08	0.23	0.31	0.38	1.08			0
21	TBG6A.1 7x8-7 TTTT	2	0	8	2	2	12	0.00	0.67	0.17	0.17	1.00			0
34	TBG6A.1 7x8-7 TTTT	2	4	6	2	4	16	0.25	0.38	0.13	0.25	1.25			0
37	TBG6A.1 7x8-7 TTTT	2	2	2	5	6	15	0.13	0.13	0.33	0.40	1.13			0
75	TBG6A.1 7x8-7 TTTT	2	1	1	2	4	8	0.13	0.13	0.25	0.50	1.13			1
77	TBG6A.1 7x8-7 TTTT	2	0	6	4	4	14	0.00	0.43	0.29	0.29	1.00			1
93	TBG6A.1 7x8-7 TTTT	2	1	2	5	3	11	0.09	0.18	0.45	0.27	1.09			1
114	TBG6A.1 7x8-7 TTTT	2	0	3	9	4	16	0.00	0.19	0.56	0.25	1.00			0
115	TBG6A.1 7x8-7 TTTT	2	3	4	6	2	15	0.20	0.27	0.40	0.13	1.20			0
117	TBG6A.1 7x8-7 TTTT	2	2	1	4	7	14	0.14	0.07	0.29	0.50	1.14			1
119	TBG6A.1 7x8-7 TTTT	2	0	3	6	4	13	0.00	0.23	0.46	0.31	1.00			0
125	TBG6A.1 7x8-7 TTTT	2	2	4	5	5	16	0.13	0.25	0.31	0.31	1.13			0
126	TBG6A.1 7x8-7 TTTT	2	2	3	3	4	12	0.17	0.25	0.25	0.33	1.17			1
156	TBG6A.1 7x8-7 TTTT	2	0	4	3	5	12	0.00	0.33	0.25	0.42	1.00			1
174	TBG6A.1 7x8-7 TTTT	2	1	4	8	1	14	0.07	0.29	0.57	0.07	1.07			1
190	TBG6A.1 7x8-7 TTTT	2	2	3	5	3	13	0.15	0.23	0.38	0.23	1.15			0
198	TBG6A.1 7x8-7 TTTT	2	0	0	7	7	14	0.00	0.00	0.50	0.50	1.00			1
209	TBG6A.1 7x8-7 TTTT	2	1	4	6	3	14	0.07	0.29	0.43	0.21	1.07			1
219	TBG6A.1 7x8-7 TTTT	2	3	4	4	1	12	0.25	0.33	0.33	0.08	1.25			1
241	TBG6A.1 7x8-7 TTTT	2	0	4	7	2	13	0.00	0.31	0.54	0.15	1.00			1
261	TBG6A.1 7x8-7 TTTT	2	0	3	9	4	16	0.00	0.19	0.56	0.25	1.00			0
282	TBG6A.1 7x8-7 TTTT	2	1	2	6	4	13	0.08	0.15	0.46	0.31	1.08			0
293	TBG6A.1 7x8-7 TTTT	2	3	2	8	2	15	0.20	0.13	0.53	0.13	1.20			0
9	TxD	1	2	1	3	7	13	0.15	0.08	0.23	0.54	1.15			

10	TxD	1	2	3	4	4	13	0.15	0.23	0.31	0.31	1.15	
13	TxD	1	0	3	9	3	15	0.00	0.20	0.60	0.20	1.00	
14	TxD	1	3	5	2	6	16	0.19	0.31	0.13	0.38	1.19	
19	TxD	1	3	3	5	2	13	0.23	0.23	0.38	0.15	1.23	
23	TxD	1	0	4	6	3	13	0.00	0.31	0.46	0.23	1.00	
28	TxD	1	3	5	4	2	14	0.21	0.36	0.29	0.14	1.21	
30	TxD	1	1	5	4	3	13	0.08	0.38	0.31	0.23	1.08	
33	TxD	1	1	5	6	2	14	0.07	0.36	0.43	0.14	1.07	
38	TxD	1	3	1	5	4	13	0.23	0.08	0.38	0.31	1.23	
43	TxD	1	2	3	3	5	13	0.15	0.23	0.23	0.38	1.15	
46	TxD	1	0	4	5	1	10	0.00	0.40	0.50	0.10	1.00	
48	TxD	1	1	4	4	1	10	0.10	0.40	0.40	0.10	1.10	
53	TxD	1	0	5	9	1	15	0.00	0.33	0.60	0.07	1.00	
56	TxD	1	1	1	6	6	14	0.07	0.07	0.43	0.43	1.07	
57	TxD	1	0	7	1	3	11	0.00	0.64	0.09	0.27	1.00	
69	TxD	1	1	2	4	7	14	0.07	0.14	0.29	0.50	1.07	
71	TxD	1	2	3	4	5	14	0.14	0.21	0.29	0.36	1.14	
74	TxD	1	0	4	10	2	16	0.00	0.25	0.63	0.13	1.00	
75	TxD	1	1	4	7	3	15	0.07	0.27	0.47	0.20	1.07	
77	TxD	1	1	7	6	1	15	0.07	0.47	0.40	0.07	1.07	
83	TxD	1	1	8	4	3	16	0.06	0.50	0.25	0.19	1.06	
105	TxD	1	2	3	6	3	14	0.14	0.21	0.43	0.21	1.14	
110	TxD	1	0	5	4	4	13	0.00	0.38	0.31	0.31	1.00	
112	TxD	1	0	3	7	4	14	0.00	0.21	0.50	0.29	1.00	
5	TBG.5Bx3B_2 DDDD	1	2	2	3	3	10	0.20	0.20	0.30	0.30	1.20	0
11	TBG.5Bx3B_2 DDDD	1	0	5	3	5	13	0.00	0.38	0.23	0.38	1.00	1
13	TBG.5Bx3B_2 DDDD	1	0	5	8	2	15	0.00	0.33	0.53	0.13	1.00	1
24	TBG.5Bx3B_2 DDDD	1	1	6	4	4	15	0.07	0.40	0.27	0.27	1.07	1
27	TBG.5Bx3B_2 DDDD	1	0	4	5	3	12	0.00	0.33	0.42	0.25	1.00	0
30	TBG.5Bx3B_2 DDDD	1	1	4	6	2	13	0.08	0.31	0.46	0.15	1.08	0
37	TBG.5Bx3B_2 DDDD	1	1	3	7	3	14	0.07	0.21	0.50	0.21	1.07	1
49	TBG.5Bx3B_2 DDDD	1	0	4	8	4	16	0.00	0.25	0.50	0.25	1.00	1
53	TBG.5Bx3B_2 DDDD	1	0	1	7	1	9	0.00	0.11	0.78	0.11	1.00	1
65	TBG.5Bx3B_2 DDDD	1	0	9	3	1	13	0.00	0.69	0.23	0.08	1.00	1
74	TBG.5Bx3B_2 DDDD	1	1	8	3	3	15	0.07	0.53	0.20	0.20	1.07	1
79	TBG.5Bx3B_2 DDDD	1	2	5	4	0	11	0.18	0.45	0.36	0.00	1.18	1
91	TBG.5Bx3B_2 DDDD	1	1	8	5	2	16	0.06	0.50	0.31	0.13	1.06	1
133	TBG.5Bx3B_2 DDDD	1	0	3	1	6	10	0.00	0.30	0.10	0.60	1.00	0
164	TBG.5Bx3B_2 DDDD	1	0	7	3	3	13	0.00	0.54	0.23	0.23	1.00	1
167	TBG.5Bx3B_2 DDDD	1	2	3	5	4	14	0.14	0.21	0.36	0.29	1.14	1
171	TBG.5Bx3B_2 DDDD	1	0	4	5	1	10	0.00	0.40	0.50	0.10	1.00	0
180	TBG.5Bx3B_2 DDDD	1	3	5	5	2	15	0.20	0.33	0.33	0.13	1.20	0
194	TBG.5Bx3B_2 DDDD	1	1	4	5	4	14	0.07	0.29	0.36	0.29	1.07	0

205	TBG.5Bx3B_2 DDDD	1	2	2	3	9	16	0.13	0.13	0.19	0.56	1.13	0
226	TBG.5Bx3B_2 DDDD	1	0	2	6	5	13	0.00	0.15	0.46	0.38	1.00	1
244	TBG.5Bx3B_2 DDDD	1	3	3	5	3	14	0.21	0.21	0.36	0.21	1.21	1
263	TBG.5Bx3B_2 DDDD	1	1	4	3	3	11	0.09	0.36	0.27	0.27	1.09	1
336	TBG.5Bx3B_2 DDDD	1	0	7	6	1	14	0.00	0.50	0.43	0.07	1.00	0
342	TBG.5Bx3B_2 DDDD	1	0	4	7	1	12	0.00	0.33	0.58	0.08	1.00	0
10	TBG.5Bx3B_4 DDDD	1	2	9	4	0	15	0.13	0.60	0.27	0.00	1.13	0
40	TBG.5Bx3B_4 DDDD	1	1	3	8	0	12	0.08	0.25	0.67	0.00	1.08	1
56	TBG.5Bx3B_4 DDDD	1	1	4	4	6	15	0.07	0.27	0.27	0.40	1.07	0
59	TBG.5Bx3B_4 DDDD	1	0	6	3	0	9	0.00	0.67	0.33	0.00	1.00	0
63	TBG.5Bx3B_4 DDDD	1	0	5	4	3	12	0.00	0.42	0.33	0.25	1.00	0
72	TBG.5Bx3B_4 DDDD	1	1	7	1	0	9	0.11	0.78	0.11	0.00	1.11	1
105	TBG.5Bx3B_4 DDDD	1	1	4	4	2	11	0.09	0.36	0.36	0.18	1.09	0
130	TBG.5Bx3B_4 DDDD	1	2	1	6	1	10	0.20	0.10	0.60	0.10	1.20	1
175	TBG.5Bx3B_4 DDDD	1	0	4	8	4	16	0.00	0.25	0.50	0.25	1.00	0
247	TBG.5Bx3B_4 DDDD	1	1	5	5	2	13	0.08	0.38	0.38	0.15	1.08	0
287	TBG.5Bx3B_4 DDDD	1	2	2	0	0	4	0.50	0.50	0.00	0.00	1.50	1
292	TBG.5Bx3B_4 DDDD	1	1	5	3	3	12	0.08	0.42	0.25	0.25	1.08	1
304	TBG.5Bx3B_4 DDDD	1	2	6	4	3	15	0.13	0.40	0.27	0.20	1.13	0
4	TBG.5Bx3B_6 DDDD	1	2	5	5	2	14	0.14	0.36	0.36	0.14	1.14	0
6	TBG.5Bx3B_6 DDDD	1	2	2	11	2	17	0.12	0.12	0.65	0.12	1.12	1
68	TBG.5Bx3B_6 DDDD	1	0	4	5	3	12	0.00	0.33	0.42	0.25	1.00	0
80	TBG.5Bx3B_6 DDDD	1	3	3	4	2	12	0.25	0.25	0.33	0.17	1.25	0
81	TBG.5Bx3B_6 DDDD	1	2	4	3	3	12	0.17	0.33	0.25	0.25	1.17	0
92	TBG.5Bx3B_6 DDDD	1	1	2	6	3	12	0.08	0.17	0.50	0.25	1.08	0
94	TBG.5Bx3B_6 DDDD	1	1	5	1	8	15	0.07	0.33	0.07	0.53	1.07	0
102	TBG.5Bx3B_6 DDDD	1	1	8	5	0	14	0.07	0.57	0.36	0.00	1.07	0
104	TBG.5Bx3B_6 DDDD	1	2	1	8	5	16	0.13	0.06	0.50	0.31	1.13	0
111	TBG.5Bx3B_6 DDDD	1	1	7	2	4	14	0.07	0.50	0.14	0.29	1.07	1
156	TBG.5Bx3B_6 DDDD	1	1	4	2	3	10	0.10	0.40	0.20	0.30	1.10	0
159	TBG.5Bx3B_6 DDDD	1	1	6	3	2	12	0.08	0.50	0.25	0.17	1.08	0
163	TBG.5Bx3B_6 DDDD	1	1	9	2	1	13	0.08	0.69	0.15	0.08	1.08	1
190	TBG.5Bx3B_6 DDDD	1	1	4	7	2	14	0.07	0.29	0.50	0.14	1.07	0
197	TBG.5Bx3B_6 DDDD	1	0	2	8	2	12	0.00	0.17	0.67	0.17	1.00	1
200	TBG.5Bx3B_6 DDDD	1	1	1	8	3	13	0.08	0.08	0.62	0.23	1.08	1
201	TBG.5Bx3B_6 DDDD	1	1	6	5	2	14	0.07	0.43	0.36	0.14	1.07	0
213	TBG.5Bx3B_6 DDDD	1	1	4	5	1	11	0.09	0.36	0.45	0.09	1.09	1
256	TBG.5Bx3B_6 DDDD	1	0	6	7	3	16	0.00	0.38	0.44	0.19	1.00	0
257	TBG.5Bx3B_6 DDDD	1	1	6	6	1	14	0.07	0.43	0.43	0.07	1.07	1
270	TBG.5Bx3B_6 DDDD	1	1	5	6	4	16	0.06	0.31	0.38	0.25	1.06	0
295	TBG.5Bx3B_6 DDDD	1	0	2	4	4	10	0.00	0.20	0.40	0.40	1.00	1
303	TBG.5Bx3B_6 DDDD	1	0	7	6	2	15	0.00	0.47	0.40	0.13	1.00	0
309	TBG.5Bx3B_6 DDDD	1	1	3	7	1	12	0.08	0.25	0.58	0.08	1.08	1

325	TBG.5Bx3B_6 DDDD	1	1	6	7	2	16	0.06	0.38	0.44	0.13	1.06	0
29	TBG5bx3b-11 DDDD	2	1	6	3	4	14	0.07	0.43	0.21	0.29	1.07	0
49	TBG5bx3b-11 DDDD	2	0	2	6	5	13	0.00	0.15	0.46	0.38	1.00	1
112	TBG5bx3b-11 DDDD	2	0	2	4	5	11	0.00	0.18	0.36	0.45	1.00	1
150	TBG5bx3b-11 DDDD	2	1	3	6	3	13	0.08	0.23	0.46	0.23	1.08	1
161	TBG5bx3b-11 DDDD	2	2	5	7	1	15	0.13	0.33	0.47	0.07	1.13	0
207	TBG5bx3b-11 DDDD	2	1	3	5	2	11	0.09	0.27	0.45	0.18	1.09	1
263	TBG5bx3b-11 DDDD	2	1	2	3	1	7	0.14	0.29	0.43	0.14	1.14	1
269	TBG5bx3b-11 DDDD	2	1	2	7	5	15	0.07	0.13	0.47	0.33	1.07	0
1	TBG 7x23_2 DDDD	2	2	4	5	4	15	0.13	0.27	0.33	0.27	1.13	0
2	TBG 7x23_2 DDDD	2	0	5	5	4	14	0.00	0.36	0.36	0.29	1.00	1
55	TBG 7x23_2 DDDD	2		3	9	1	13	0.00	0.23	0.69	0.08	1.00	0
57	TBG 7x23_2 DDDD	2	2	3	5	5	15	0.13	0.20	0.33	0.33	1.13	1
65	TBG 7x23_2 DDDD	2	0	4	5	2	11	0.00	0.36	0.45	0.18	1.00	1
82	TBG 7x23_2 DDDD	2	1	4	6	3	14	0.07	0.29	0.43	0.21	1.07	1
88	TBG 7x23_2 DDDD	2	0	7	2	7	16	0.00	0.44	0.13	0.44	1.00	1
108	TBG 7x23_2 DDDD	2	2	3	6	1	12	0.17	0.25	0.50	0.08	1.17	0
116	TBG 7x23_2 DDDD	2	0	4	4	4	12	0.00	0.33	0.33	0.33	1.00	0
138	TBG 7x23_2 DDDD	2	0	6	7	2	15	0.00	0.40	0.47	0.13	1.00	1
168	TBG 7x23_2 DDDD	2	0	3	9	1	13	0.00	0.23	0.69	0.08	1.00	1
173	TBG 7x23_2 DDDD	2	2	2	9	1	14	0.14	0.14	0.64	0.07	1.14	1
177	TBG 7x23_2 DDDD	2	0	3	11	2	16	0.00	0.19	0.69	0.13	1.00	0
189	TBG 7x23_2 DDDD	2	0	5	9	2	16	0.00	0.31	0.56	0.13	1.00	0
192	TBG 7x23_2 DDDD	2	2	2	9	3	16	0.13	0.13	0.56	0.19	1.13	0
220	TBG 7x23_2 DDDD	2	1	4	5	1	11	0.09	0.36	0.45	0.09	1.09	1
221	TBG 7x23_2 DDDD	2	1	4	8	1	14	0.07	0.29	0.57	0.07	1.07	1
225	TBG 7x23_2 DDDD	2	2	3	8	3	16	0.13	0.19	0.50	0.19	1.13	0
229	TBG 7x23_2 DDDD	2	1	4	8	0	13	0.08	0.31	0.62	0.00	1.08	0
255	TBG 7x23_2 DDDD	2	0	1	10	2	13	0.00	0.08	0.77	0.15	1.00	1
256	TBG 7x23_2 DDDD	2	2	2	8	2	14	0.14	0.14	0.57	0.14	1.14	0
259	TBG 7x23_2 DDDD	2	0	6	4	3	13	0.00	0.46	0.31	0.23	1.00	1
266	TBG 7x23_2 DDDD	2	1	3	7	0	11	0.09	0.27	0.64	0.00	1.09	0
280	TBG 7x23_2 DDDD	2	0	1	11	3	15	0.00	0.07	0.73	0.20	1.00	0
290	TBG 7x23_2 DDDD	2	2	1	3	6	12	0.17	0.08	0.25	0.50	1.17	1
5	TBG 7x23_3 DDDD	2	0	3	5	3	11	0.00	0.27	0.45	0.27	1.00	1
19	TBG 7x23_3 DDDD	2	1	1	7	6	15	0.07	0.07	0.47	0.40	1.07	1
25	TBG 7x23_3 DDDD	2	0	2	7	1	10	0.00	0.20	0.70	0.10	1.00	0
26	TBG 7x23_3 DDDD	2	1	7	3	2	13	0.08	0.54	0.23	0.15	1.08	0
38	TBG 7x23_3 DDDD	2	1	6	6	3	16	0.06	0.38	0.38	0.19	1.06	1
54	TBG 7x23_3 DDDD	2	1	4	4	3	12	0.08	0.33	0.33	0.25	1.08	1
60	TBG 7x23_3 DDDD	2	0	3	6	5	14	0.00	0.21	0.43	0.36	1.00	1
69	TBG 7x23_3 DDDD	2	0	5	6	1	12	0.00	0.42	0.50	0.08	1.00	1
94	TBG 7x23_3 DDDD	2	0	6	6	1	13	0.00	0.46	0.46	0.08	1.00	0

136	TBG 7x23_3 DDDD	2	1	5	6	3	15	0.07	0.33	0.40	0.20	1.07	0
159	TBG 7x23_3 DDDD	2	1	6	4	3	14	0.07	0.43	0.29	0.21	1.07	0
179	TBG 7x23_3 DDDD	2	0	4	9	1	14	0.00	0.29	0.64	0.07	1.00	0
188	TBG 7x23_3 DDDD	2	1	5	6	4	16	0.06	0.31	0.38	0.25	1.06	1
199	TBG 7x23_3 DDDD	2	2	9	3	2	16	0.13	0.56	0.19	0.13	1.13	0
205	TBG 7x23_3 DDDD	2	5	2	7	1	15	0.33	0.13	0.47	0.07	1.33	1
230	TBG 7x23_3 DDDD	2	1	3	3	2	9	0.11	0.33	0.33	0.22	1.11	0
231	TBG 7x23_3 DDDD	2	1	0	7	3	11	0.09	0.00	0.64	0.27	1.09	1
240	TBG 7x23_3 DDDD	2	0	6	6	3	15	0.00	0.40	0.40	0.20	1.00	1
253	TBG 7x23_3 DDDD	2	3	4	5	1	13	0.23	0.31	0.38	0.08	1.23	0
257	TBG 7x23_3 DDDD	2	1	4	8	2	15	0.07	0.27	0.53	0.13	1.07	0
258	TBG 7x23_3 DDDD	2	2	5	4	3	14	0.14	0.36	0.29	0.21	1.14	1
268	TBG 7x23_3 DDDD	2	0	3	10	1	14	0.00	0.21	0.71	0.07	1.00	0
276	TBG 7x23_3 DDDD	2	2	3	6	3	14	0.14	0.21	0.43	0.21	1.14	1
281	TBG 7x23_3 DDDD	2	1	4	9	1	15	0.07	0.27	0.60	0.07	1.07	0
289	TBG 7x23_3 DDDD	2	2	1	11	1	15	0.13	0.07	0.73	0.07	1.13	0
9	TBG 7x23_4 DDDD	2	1	4	5	3	13	0.08	0.31	0.38	0.23	1.08	1
12	TBG 7x23_4 DDDD	2	3	2	6	4	15	0.20	0.13	0.40	0.27	1.20	1
32	TBG 7x23_4 DDDD	2	0	2	2	5	9	0.00	0.22	0.22	0.56	1.00	0
35	TBG 7x23_4 DDDD	2	0	5	5	4	14	0.00	0.36	0.36	0.29	1.00	0
36	TBG 7x23_4 DDDD	2	0	3	10	2	15	0.00	0.20	0.67	0.13	1.00	1
45	TBG 7x23_4 DDDD	2	3	2	4	5	14	0.21	0.14	0.29	0.36	1.21	1
50	TBG 7x23_4 DDDD	2	1	3	6	2	12	0.08	0.25	0.50	0.17	1.08	0
62	TBG 7x23_4 DDDD	2	0	3	6	1	10	0.00	0.30	0.60	0.10	1.00	0
71	TBG 7x23_4 DDDD	2	1	2	10	1	14	0.07	0.14	0.71	0.07	1.07	0
86	TBG 7x23_4 DDDD	2	1	3	1	5	10	0.10	0.30	0.10	0.50	1.10	0
100	TBG 7x23_4 DDDD	2	1	0	5	7	13	0.08	0.00	0.38	0.54	1.08	0
129	TBG 7x23_4 DDDD	2	0	4	6	4	14	0.00	0.29	0.43	0.29	1.00	1
137	TBG 7x23_4 DDDD	2	0	4	7	5	16	0.00	0.25	0.44	0.31	1.00	0
145	TBG 7x23_4 DDDD	2	1	8	3	2	14	0.07	0.57	0.21	0.14	1.07	0
148	TBG 7x23_4 DDDD	2	2	2	6	5	15	0.13	0.13	0.40	0.33	1.13	1
149	TBG 7x23_4 DDDD	2	0	2	9	0	11	0.00	0.18	0.82	0.00	1.00	0
153	TBG 7x23_4 DDDD	2	2	6	6	1	15	0.13	0.40	0.40	0.07	1.13	0
158	TBG 7x23_4 DDDD	2	1	3	7	3	14	0.07	0.21	0.50	0.21	1.07	0
171	TBG 7x23_4 DDDD	2	1	5	6	2	14	0.07	0.36	0.43	0.14	1.07	0
175	TBG 7x23_4 DDDD	2	0	0	7	3	10	0.00	0.00	0.70	0.30	1.00	0
195	TBG 7x23_4 DDDD	2	0	3	7	5	15	0.00	0.20	0.47	0.33	1.00	1
211	TBG 7x23_4 DDDD	2	0	1	10	4	15	0.00	0.07	0.67	0.27	1.00	0
245	TBG 7x23_4 DDDD	2	1	4	5	3	13	0.08	0.31	0.38	0.23	1.08	1
271	TBG 7x23_4 DDDD	2	0	1	9	3	13	0.00	0.08	0.69	0.23	1.00	0
273	TBG 7x23_4 DDDD	2	1	3	8	1	13	0.08	0.23	0.62	0.08	1.08	0
11	TBG 7x23_5 DDDD	2	1	1	4	5	11	0.09	0.09	0.36	0.45	1.09	1
18	TBG 7x23_5 DDDD	2	0	9	2	3	14	0.00	0.64	0.14	0.21	1.00	1

20	TBG 7x23_5 DDDD	2	1	2	7	2	12	0.08	0.17	0.58	0.17	1.08	0
31	TBG 7x23_5 DDDD	2	0	5	4	5	14	0.00	0.36	0.29	0.36	1.00	0
41	TBG 7x23_5 DDDD	2	2	6	2	4	14	0.14	0.43	0.14	0.29	1.14	1
52	TBG 7x23_5 DDDD	2	3	4	6	3	16	0.19	0.25	0.38	0.19	1.19	1
56	TBG 7x23_5 DDDD	2	2	3	5	1	11	0.18	0.27	0.45	0.09	1.18	0
58	TBG 7x23_5 DDDD	2	0	4	5	0	9	0.00	0.44	0.56	0.00	1.00	1
74	TBG 7x23_5 DDDD	2	4	4	4	2	14	0.29	0.29	0.29	0.14	1.29	0
92	TBG 7x23_5 DDDD	2	0	5	7	3	15	0.00	0.33	0.47	0.20	1.00	0
105	TBG 7x23_5 DDDD	2	0	4	8	3	15	0.00	0.27	0.53	0.20	1.00	0
109	TBG 7x23_5 DDDD	2	3	4	3	3	13	0.23	0.31	0.23	0.23	1.23	1
118	TBG 7x23_5 DDDD	2	0	6	7	2	15	0.00	0.40	0.47	0.13	1.00	0
121	TBG 7x23_5 DDDD	2	0	5	6	2	13	0.00	0.38	0.46	0.15	1.00	0
130	TBG 7x23_5 DDDD	2	2	3	4	3	12	0.17	0.25	0.33	0.25	1.17	0
133	TBG 7x23_5 DDDD	2	0	5	6	4	15	0.00	0.33	0.40	0.27	1.00	0
140	TBG 7x23_5 DDDD	2	2	3	8	1	14	0.14	0.21	0.57	0.07	1.14	0
144	TBG 7x23_5 DDDD	2	0	3	7	2	12	0.00	0.25	0.58	0.17	1.00	0
176	TBG 7x23_5 DDDD	2	1	4	5	2	12	0.08	0.33	0.42	0.17	1.08	0
204	TBG 7x23_5 DDDD	2	0	3	11	2	16	0.00	0.19	0.69	0.13	1.00	1
222	TBG 7x23_5 DDDD	2	0	5	9	2	16	0.00	0.31	0.56	0.13	1.00	1
227	TBG 7x23_5 DDDD	2	0	3	7	3	13	0.00	0.23	0.54	0.23	1.00	0
233	TBG 7x23_5 DDDD	2	1	5	6	1	13	0.08	0.38	0.46	0.08	1.08	0
292	TBG 7x23_5 DDDD	2	0	3	4	3	10	0.00	0.30	0.40	0.30	1.00	1
297	TBG 7x23_5 DDDD	2	0	6	9	0	15	0.00	0.40	0.60	0.00	1.00	0

Table S1 notes: Primary phenotyping of metaphase spreads from diploids and tetraploids segregating for *ASY1-D* and *ASY1-T*; tetraploids are pooled from two experiments that do not differ statistically (Chi-squared test, p-value = 0.27). Image numbers correspond to images provided under https://doi.org/10.3929/ethz-b-000386103. Genotype indicates individual name as well as genotype class. Exp = experiment number. Trait definitions as in Figure 2, with left columns giving raw numbers and right columns giving proportion of each shape among scorable bivalents. o = ring (2 crossover) bivalents; + = proportion bivalents with substantial central chromatin past the chiasma; | = proportion bivalents with very little central chromatin past the chiasma; ł = bivalents intermediate between | and +. Ts = total scorable bivalents in each cell. Proportions of bivalents are given as a proportion of each type out of the total scorable bivalents. CO/Bv = crossover frequency per bivalent, calculated as: (2(O)+sum(I, ł, +)) / Ts. Mv = multivalents assayed as presence (1) or absence (0).

Table S2: Chi-squared test results for bivalent shape comparisons of diploids, and tetraploids segregating D vs T alleles of ASY1

Comparison	p-value
All genotypes	**2.20 x 10^{-16}**
**Comparison**	**Bonferroni adjusted p-value**
TTTT vs DDDD	**3.67 x 10^{-07}**
TTTT vs TxD	**0.73**
TTTT vs Diploid (x2)	**1.15 x 10^{-13}**
DDDD vs TxD	**0.69**
DDDD vs Diploid (x2)	**1.83 x 10^{-09}**
TxD vs Diploid (x2)	**2.17 x 10^{-07}**

Fisher test result for multivalent presence rates

Comparison	p-value
TTTT vs DDDD	**0.031**

Table S2 notes: Results from post-hoc pairwise chi-squared tests with Bonferroni correction to determine if significant differences occurred between individual pairs of genotypes using bivalent count data from Table S1. For multivalent presence/absence data we used Fishers exact test comparing the DDDD and TTTT genotypes only.

Table S3: Estimated trait means (number per cell) of different bivalent shapes and p-values from GLMMs for ASY1 D vs T

**Rod**			
	Est mean	2.5% Conf	97.5% Conf
Diploid (x2) (1 plant, 25 cells)	2.63	1.87	3.71
DDDD (8 plants, 170 cells)	2.63	2.31	2.99
TXD (1 plants, 25 cells)	3.39	2.45	4.68
TTTT (4 plants, 100 cells)	4.21	3.6	4.91

Rod p values			
	Dip(x2)	DDDD	TXD
Dip(x2)			
DDDD	0.9974		
TXD	0.2917	0.152	
TTTT	0.0143	0.0000044	0.236

**Cross**			
	Est mean	2.5% Conf	97.5% Conf
Diploid (x2) (1 plant, 25 cells)	6.15	4.81	7.86
DDDD (8 plants, 170 cells)	3.94	3.56	4.37
TXD (1 plants, 25 cells)	3.91	2.98	5.14
TTTT (4 plants, 100 cells)	3.02	2.61	3.5

Cross p values			
	Dip(x2)	DDDD	TXD
Dip(x2)			
DDDD	0.00106		
TXD	0.01568	0.95368	
TTTT	0.0000011	0.00346	0.1033

**Ring**			
	Est mean	2.5% Conf	97.5% Conf
Diploid (x2) (1 plant, 25 cells)	2.56	1.97	3.32
DDDD (8 plants, 170 cells)	0.92	0.79	1.08
TXD (1 plants, 25 cells)	1.2	0.83	1.73
TTTT (4 plants, 100 cells)	1.54	1.3	1.81

Ring p values			
	Dip(x2)	DDDD	TXD
Dip(x2)			
DDDD	7.33E-11		
TXD	0.00104	0.203	
TTTT	0.00127	0.0000128	0.22716

**Bowtie**			
	Est mean	2.5% Conf	97.5% Conf
Diploid (x2) (1 plant, 25 cells)	3.19	2.46	4.14
DDDD (8 plants, 170 cells)	5.66	5.21	6.14
TXD (1 plants, 25 cells)	5.11	4.09	6.39
TTTT (4 plants, 100 cells)	4.78	4.26	5.36

Bowtie p values			
	Dip(x2)	DDDD	TXD
Dip(x2)			
DDDD	0.0000411		
TXD	0.00721	0.4042	
TTTT	0.00545	0.0187	0.59911

**MV**			
	Est mean	2.5% Conf	97.5% Conf
DDDD (8 plants, 170 cells)	0.491	0.409	0.574
TTTT (4 plants, 100 cells)	0.349	0.253	0.458

Multivalent p value	
	DDDD
TTTT	0.0416

Table S3 notes: Trait means (left) and p-values (right) calculated from GLMM modeling. Normal approximations of 95% confidence intervals (range given from 2.5% Conf to 97.5% Conf) were calculated as 1.96 estimated standard errors.

Table S4: Cytological trait values for *A. arenosa* tetraploids segregating ASY1 and ASY3 – experiment 1

								Proportions (out of total scorable bivalents)					
**Gen**	**Plant**	**Image**	**O**	**+**	**ł**	**I**	**Ts**	**O**	**+**	**ł**	**I**	**CO/Bv**	**MV**
DD	91	8	3	2	6	2	13	0.15	0.46	0.15	0.23	1.23	1
DD	91	10	3	2	6	1	12	0.08	0.50	0.17	0.25	1.25	0
DD	91	12	3	2	8	1	14	0.07	0.57	0.14	0.21	1.21	1
DD	91	14	3	2	7	3	15	0.20	0.47	0.13	0.20	1.20	1
DD	91	17	2	0	8	3	13	0.23	0.62	0.00	0.15	1.15	1
DD	91	19	3	0	8	2	13	0.15	0.62	0.00	0.23	1.23	1
DD	91	20	3	3	4	0	10	0.00	0.40	0.30	0.30	1.30	0
DD	91	21	4	2	9	1	16	0.06	0.56	0.13	0.25	1.25	0
DD	91	22	1	1	8	0	10	0.00	0.80	0.10	0.10	1.10	1
DD	91	25	2	4	9	1	16	0.06	0.56	0.25	0.13	1.13	0
DD	91	26	2	1	5	4	12	0.33	0.42	0.08	0.17	1.17	1
DD	91	27	0	3	5	2	10	0.20	0.50	0.30	0.00	1.00	0
DD	91	29	2	2	10	0	14	0.00	0.71	0.14	0.14	1.14	1
DD	91	32	3	1	7	2	13	0.15	0.54	0.08	0.23	1.23	1
DD	91	35	3	3	5	4	15	0.27	0.33	0.20	0.20	1.20	0
DD	1T-14	1	2	0	6	2	10	0.20	0.60	0.00	0.20	1.20	0
DD	1T-14	2	4	1	5	4	14	0.29	0.36	0.07	0.29	1.29	1
DD	1T-14	3	3	1	4	2	10	0.20	0.40	0.10	0.30	1.30	1
DD	1T-14	4	2	1	7	3	13	0.23	0.54	0.08	0.15	1.15	0
DD	1T-14	5	3	1	4	2	10	0.20	0.40	0.10	0.30	1.30	0
DD	1T-14	6	1	0	8	2	11	0.18	0.73	0.00	0.09	1.09	0
DD	1T-14	8	5	3	3	2	13	0.15	0.23	0.23	0.38	1.38	0
DD	1T-14	7	3	1	4	2	10	0.20	0.40	0.10	0.30	1.30	0
DD	1T-37	9	2	2	8	3	15	0.20	0.53	0.13	0.13	1.13	0
DD	1T-37	10	0	4	8	1	13	0.08	0.62	0.31	0.00	1.00	0
DD	1T-37	12	1	1	7	1	10	0.10	0.70	0.10	0.10	1.10	0
DD	1T-37	14	1	2	8	1	12	0.08	0.67	0.17	0.08	1.08	0
DD	1T-37	22	2	1	8	4	15	0.27	0.53	0.07	0.13	1.13	0
DD	1T-37	30	0	1	8	3	12	0.25	0.67	0.08	0.00	1.00	0
DD	1T-37	32	2	0	9	2	13	0.15	0.69	0.00	0.15	1.15	1
DD	1T-37	35	1	1	7	2	11	0.18	0.64	0.09	0.09	1.09	0
DD	1T-37	36	2	1	5	4	12	0.33	0.42	0.08	0.17	1.17	0
DD	1T-37	40	1	6	5	0	12	0.00	0.42	0.50	0.08	1.08	0
DD	1T-37	47	6	2	6	1	15	0.07	0.40	0.13	0.40	1.40	1
DD	1T-37	50	1	1	10	1	13	0.08	0.77	0.08	0.08	1.08	1
DD	1T-37	54	3	1	5	4	13	0.31	0.38	0.08	0.23	1.23	1
DD	1T-37	55	3	2	8	3	16	0.19	0.50	0.13	0.19	1.19	0
DD	1T-37	56	1	3	5	2	11	0.18	0.45	0.27	0.09	1.09	1
DT	34	1	3	0	5	4	12	0.33	0.42	0.00	0.25	1.25	1

DT	34	9	2	0	10	1	13	0.08	0.77	0.00	0.15	1.15	1
DT	34	11	1	1	3	9	14	0.64	0.21	0.07	0.07	1.07	1
DT	34	13	5	1	6	3	15	0.20	0.40	0.07	0.33	1.33	0
DT	34	21	4	1	5	4	14	0.29	0.36	0.07	0.29	1.29	0
DT	34	25	3	0	7	1	11	0.09	0.64	0.00	0.27	1.27	1
DT	34	27	4	1	6	1	12	0.08	0.50	0.08	0.33	1.33	1
DT	34	33	1	1	8	3	13	0.23	0.62	0.08	0.08	1.08	1
DT	34	39	1	0	10	3	14	0.21	0.71	0.00	0.07	1.07	0
DT	34	40	6	1	5	2	14	0.14	0.36	0.07	0.43	1.43	0
DT	34	41	8	1	5	1	15	0.07	0.33	0.07	0.53	1.53	0
DT	34	42	3	0	7	6	16	0.38	0.44	0.00	0.19	1.19	1
DT	34	45	2	3	6	4	15	0.27	0.40	0.20	0.13	1.13	1
DT	34	48	3	1	7	1	12	0.08	0.58	0.08	0.25	1.25	0
DT	34	58	3	1	2	6	12	0.50	0.17	0.08	0.25	1.25	1
DT	1T-5	5	3	1	6	4	14	0.29	0.43	0.07	0.21	1.21	0
DT	1T-5	12	3	0	8	1	12	0.08	0.67	0.00	0.25	1.25	1
DT	1T-5	3	1	3	7	1	12	0.08	0.58	0.25	0.08	1.08	0
DT	1T-5	675	2	2	8	1	13	0.08	0.62	0.15	0.15	1.15	0
DT	1T-5	677	1	4	7	2	14	0.14	0.50	0.29	0.07	1.07	0
DT	1T-5	676	3	0	8	1	12	0.08	0.67	0.00	0.25	1.25	1
DT	1T-5	18	1	2	7	0	10	0.00	0.70	0.20	0.10	1.10	0
DT	1T-5	32	2	1	8	3	14	0.21	0.57	0.07	0.14	1.14	1
DT	1T-5	7	1	1	7	2	11	0.18	0.64	0.09	0.09	1.09	1
DT	1T-5	8	2	1	7	1	11	0.09	0.64	0.09	0.18	1.18	0
DT	1T-5	10	3	1	7	2	13	0.15	0.54	0.08	0.23	1.23	1
DT	1T-5	674	1	1	10	1	13	0.08	0.77	0.08	0.08	1.08	0
DT	1T-5	681	2	3	5	2	12	0.17	0.42	0.25	0.17	1.17	0
DT	1T-5	15	3	0	9	3	15	0.20	0.60	0.00	0.20	1.20	0
DT	1T-5	2	2	2	5	2	11	0.18	0.45	0.18	0.18	1.18	0
DT	4T-15	2	2	4	7	1	14	0.07	0.50	0.29	0.14	1.14	0
DT	4T-15	3	4	3	6	3	16	0.19	0.38	0.19	0.25	1.25	0
DT	4T-15	4	5	2	4	2	13	0.15	0.31	0.15	0.38	1.38	1
DT	4T-15	28	1	1	8	2	12	0.17	0.67	0.08	0.08	1.08	0
DT	4T-15	60.1	3	3	4	5	15	0.33	0.27	0.20	0.20	1.20	0
DT	4T-15	64	6	1	5	2	14	0.14	0.36	0.07	0.43	1.43	0
DT	4T-15	66	4	2	4	4	14	0.29	0.29	0.14	0.29	1.29	0
DT	4T-15	67	4	2	7	0	13	0.00	0.54	0.15	0.31	1.31	1
DT	4T-15	70	5	1	6	1	13	0.08	0.46	0.08	0.38	1.38	1
DT	4T-15	71	2	1	10	1	14	0.07	0.71	0.07	0.14	1.14	1
DT	4T-15	72	4	3	4	2	13	0.15	0.31	0.23	0.31	1.31	1
DT	4T-15	73	7	2	8	0	17	0.00	0.47	0.12	0.41	1.41	0
DT	4T-15	76	4	2	6	3	15	0.20	0.40	0.13	0.27	1.27	0
DT	4T-15	79	4	1	6	1	12	0.08	0.50	0.08	0.33	1.33	0

DT	4T-15	85	3	3	10	0	16	0.00	0.63	0.19	0.19	1.19	1
DT	5T-4	1	4	1	6	4	15	0.27	0.40	0.07	0.27	1.27	1
DT	5T-4	3	3	0	7	1	11	0.09	0.64	0.00	0.27	1.27	1
DT	5T-4	9	2	1	6	3	12	0.25	0.50	0.08	0.17	1.17	1
DT	5T-4	12	1	0	10	1	12	0.08	0.83	0.00	0.08	1.08	1
DT	5T-4	17	2	1	7	1	11	0.09	0.64	0.09	0.18	1.18	1
DT	5T-4	19	0	3	3	3	9	0.33	0.33	0.33	0.00	1.00	0
DT	5T-4	20	2	3	3	5	13	0.38	0.23	0.23	0.15	1.15	1
DT	5T-4	21	6	2	3	1	12	0.08	0.25	0.17	0.50	1.50	1
DT	5T-4	22	1	1	7	2	11	0.18	0.64	0.09	0.09	1.09	1
DT	5T-4	23	1	1	8	0	10	0.00	0.80	0.10	0.10	1.10	1
DT	5T-4	27	1	3	6	1	11	0.09	0.55	0.27	0.09	1.09	0
DT	5T-4	29	3	2	7	2	14	0.14	0.50	0.14	0.21	1.21	0
DT	5T-4	30	4	3	6	1	14	0.07	0.43	0.21	0.29	1.29	0
DT	5T-4	33	2	3	3	2	10	0.20	0.30	0.30	0.20	1.20	0
DT	5T-4	38	1	2	8	4	15	0.27	0.53	0.13	0.07	1.07	1
DT	6T-11	1	1	1	7	2	11	0.18	0.64	0.09	0.09	1.09	0
DT	6T-11	4	5	0	8	2	15	0.13	0.53	0.00	0.33	1.33	0
DT	6T-11	5	2	3	7	3	15	0.20	0.47	0.20	0.13	1.13	0
DT	6T-11	7	3	1	9	2	15	0.13	0.60	0.07	0.20	1.20	1
DT	6T-11	9	4	1	6	2	13	0.15	0.46	0.08	0.31	1.31	0
DT	6T-11	11	5	1	8	1	15	0.07	0.53	0.07	0.33	1.33	0
DT	6T-11	13	1	0	9	2	12	0.17	0.75	0.00	0.08	1.08	0
DT	6T-11	14	2	4	3	2	11	0.18	0.27	0.36	0.18	1.18	0
DT	6T-11	28	1	2	9	1	13	0.08	0.69	0.15	0.08	1.08	0
DT	6T-11	16	7	1	5	2	15	0.13	0.33	0.07	0.47	1.47	0
DT	6T-11	18	1	0	5	4	10	0.40	0.50	0.00	0.10	1.10	0
DT	6T-11	19	2	3	7	0	12	0.00	0.58	0.25	0.17	1.17	0
DT	6T-11	21	2	2	9	0	13	0.00	0.69	0.15	0.15	1.15	1
DT	6T-11	22	0	3	8	1	12	0.08	0.67	0.25	0.00	1.00	1
DT	6T-11	26	1	3	8	2	14	0.14	0.57	0.21	0.07	1.07	0
TD	143	1	5	0	4	2	11	0.18	0.36	0.00	0.45	1.45	0
TD	143	2	2	0	8	1	11	0.09	0.73	0.00	0.18	1.18	1
TD	143	3	3	1	8	3	15	0.20	0.53	0.07	0.20	1.20	0
TD	143	4	1	2	12	0	15	0.00	0.80	0.13	0.07	1.07	1
TD	143	5	1	1	8	3	13	0.23	0.62	0.08	0.08	1.08	0
TD	143	6	1	2	8	0	11	0.00	0.73	0.18	0.09	1.09	1
TD	143	7	3	0	8	1	12	0.08	0.67	0.00	0.25	1.25	1
TD	143	8	3	4	4	1	12	0.08	0.33	0.33	0.25	1.25	0
TD	143	9.1	3	1	6	6	16	0.38	0.38	0.06	0.19	1.19	0
TD	143	9.2	7	0	5	4	16	0.25	0.31	0.00	0.44	1.44	0
TD	143	10	1	1	7	2	11	0.18	0.64	0.09	0.09	1.09	0
TD	143	11	10	1	2	1	14	0.07	0.14	0.07	0.71	1.71	0

TD	143	13	4	3	2	4	13	0.31	0.15	0.23	0.31	1.31	0
TD	143	14	3	1	6	1	11	0.09	0.55	0.09	0.27	1.27	0
TD	1T-17	1	4	1	10	0	15	0.00	0.67	0.07	0.27	1.27	0
TD	1T-17	3	3	0	4	4	11	0.36	0.36	0.00	0.27	1.27	1
TD	1T-17	10	2	2	8	2	14	0.14	0.57	0.14	0.14	1.14	1
TD	1T-17	13	1	0	11	0	12	0.00	0.92	0.00	0.08	1.08	1
TD	1T-17	14	4	1	9	0	14	0.00	0.64	0.07	0.29	1.29	0
TD	1T-17	15	3	1	9	1	14	0.07	0.64	0.07	0.21	1.21	0
TD	1T-17	16	1	0	12	2	15	0.13	0.80	0.00	0.07	1.07	0
TD	1T-17	17	0	4	6	1	11	0.09	0.55	0.36	0.00	1.00	1
TD	1T-17	20	2	2	7	3	14	0.21	0.50	0.14	0.14	1.14	0
TD	1T-17	23	2	3	6	1	12	0.08	0.50	0.25	0.17	1.17	1
TD	1T-17	28	2	1	8	2	13	0.15	0.62	0.08	0.15	1.15	0
TD	1T-17	30	2	1	7	2	12	0.17	0.58	0.08	0.17	1.17	0
TD	1T-17	35	2	3	3	4	12	0.33	0.25	0.25	0.17	1.17	1
TD	1T-17	36	4	0	7	3	14	0.21	0.50	0.00	0.29	1.29	1
TD	1T-17	37	0	1	8	5	14	0.36	0.57	0.07	0.00	1.00	1
TD	2T-19	1	2	0	7	3	12	0.25	0.58	0.00	0.17	1.17	0
TD	2T-19	2	3	1	4	2	10	0.20	0.40	0.10	0.30	1.30	1
TD	2T-19	3	2	2	7	3	14	0.21	0.50	0.14	0.14	1.14	1
TD	2T-19	4	2	0	8	2	12	0.17	0.67	0.00	0.17	1.17	1
TD	2T-19	5	6	1	2	3	12	0.25	0.17	0.08	0.50	1.50	0
TD	2T-19	6	3	1	8	3	15	0.20	0.53	0.07	0.20	1.20	0
TD	2T-19	7	7	1	6	0	14	0.00	0.43	0.07	0.50	1.50	1
TD	2T-19	9	5	1	4	4	14	0.29	0.29	0.07	0.36	1.36	0
TD	2T-19	11	4	0	7	2	13	0.15	0.54	0.00	0.31	1.31	1
TD	2T-19	12	4	0	6	1	11	0.09	0.55	0.00	0.36	1.36	0
TD	2T-19	15	2	1	2	6	11	0.55	0.18	0.09	0.18	1.18	1
TD	2T-19	20	3	0	6	2	11	0.18	0.55	0.00	0.27	1.27	1
TD	2T-19	21	4	1	10	0	15	0.00	0.67	0.07	0.27	1.27	0
TD	2T-19	22	4	0	6	4	14	0.29	0.43	0.00	0.29	1.29	0
TD	2T-19	25	4	1	8	1	14	0.07	0.57	0.07	0.29	1.29	0
TD	2T-25	1	3	0	7	4	14	0.29	0.50	0.00	0.21	1.21	1
TD	2T-25	2	1	0	7	5	13	0.38	0.54	0.00	0.08	1.08	1
TD	2T-25	4	3	0	6	2	11	0.18	0.55	0.00	0.27	1.27	0
TD	2T-25	7	3	1	9	1	14	0.07	0.64	0.07	0.21	1.21	1
TD	2T-25	8	2	3	7	3	15	0.20	0.47	0.20	0.13	1.13	0
TD	2T-25	10	2	2	9	2	15	0.13	0.60	0.13	0.13	1.13	1
TD	2T-25	11	4	1	7	2	14	0.14	0.50	0.07	0.29	1.29	0
TD	2T-37	2	1	1	10	3	15	0.20	0.67	0.07	0.07	1.07	1
TD	2T-37	3	1	1	6	4	12	0.33	0.50	0.08	0.08	1.08	0
TD	2T-37	4	1	3	5	3	12	0.25	0.42	0.25	0.08	1.08	1
TD	2T-37	5	2	5	7	1	15	0.07	0.47	0.33	0.13	1.13	0

TD	2T-37	6	6	1	1	2	10	0.20	0.10	0.10	0.60	1.60	1
TD	2T-37	8	0	2	8	5	15	0.33	0.53	0.13	0.00	1.00	0
TD	2T-37	9	1	2	5	5	13	0.38	0.38	0.15	0.08	1.08	0
TD	2T-37	10	0	1	7	4	12	0.33	0.58	0.08	0.00	1.00	1
TD	2T-37	11	1	2	7	1	11	0.09	0.64	0.18	0.09	1.09	0
TD	2T-37	12	4	0	4	4	12	0.33	0.33	0.00	0.33	1.33	1
TD	2T-37	13	0	2	7	2	11	0.18	0.64	0.18	0.00	1.00	0
TD	2T-37	14	0	2	7	3	12	0.25	0.58	0.17	0.00	1.00	1
TD	2T-37	15	1	2	7	4	14	0.29	0.50	0.14	0.07	1.07	0
TD	2T-37	17	3	1	7	4	15	0.27	0.47	0.07	0.20	1.20	0
TD	2T-37	21	0	3	6	2	11	0.18	0.55	0.27	0.00	1.00	1
TD	3T-16	2	3	2	7	1	13	0.08	0.54	0.15	0.23	1.23	1
TD	3T-16	7	2	2	5	4	13	0.31	0.38	0.15	0.15	1.15	1
TD	3T-16	25	3	1	6	2	12	0.17	0.50	0.08	0.25	1.25	1
TD	3T-16	27	5	0	8	0	13	0.00	0.62	0.00	0.38	1.38	1
TD	3T-16	35	4	1	8	1	14	0.07	0.57	0.07	0.29	1.29	1
TD	3T-16	41	3	0	6	5	14	0.36	0.43	0.00	0.21	1.21	0
TD	3T-16	44	3	1	6	3	13	0.23	0.46	0.08	0.23	1.23	1
TD	3T-16	47	2	1	7	2	12	0.17	0.58	0.08	0.17	1.17	0
TD	3T-16	51	2	1	6	3	12	0.25	0.50	0.08	0.17	1.17	1
TD	3T-16	52	1	1	7	5	14	0.36	0.50	0.07	0.07	1.07	0
TD	3T-16	63	1	1	9	1	12	0.08	0.75	0.08	0.08	1.08	1
TD	3T-16	64	1	2	9	2	14	0.14	0.64	0.14	0.07	1.07	1
TD	3T-16	76	3	1	7	1	12	0.08	0.58	0.08	0.25	1.25	1
TD	3T-16	81	1	0	9	3	13	0.23	0.69	0.00	0.08	1.08	0
TD	3T-16	82	2	1	7	2	12	0.17	0.58	0.08	0.17	1.17	1
TT	151	3	4	1	6	2	13	0.15	0.46	0.08	0.31	1.31	1
TT	151	5	4	1	3	2	10	0.20	0.30	0.10	0.40	1.40	1
TT	151	7	2	2	5	2	11	0.18	0.45	0.18	0.18	1.18	1
TT	151	8	2	3	6	1	12	0.08	0.50	0.25	0.17	1.17	1
TT	151	9	4	0	9	0	13	0.00	0.69	0.00	0.31	1.31	0
TT	151	10	2	1	6	4	13	0.31	0.46	0.08	0.15	1.15	0
TT	151	16	4	1	4	2	11	0.18	0.36	0.09	0.36	1.36	1
TT	151	18.2	2	2	6	3	13	0.23	0.46	0.15	0.15	1.15	1
TT	151	19	4	2	5	1	12	0.08	0.42	0.17	0.33	1.33	1
TT	151	21	4	0	4	3	11	0.27	0.36	0.00	0.36	1.36	0
TT	151	24	4	1	4	3	12	0.25	0.33	0.08	0.33	1.33	0
TT	151	26	3	1	7	2	13	0.15	0.54	0.08	0.23	1.23	0
TT	151	27	4	0	8	2	14	0.14	0.57	0.00	0.29	1.29	1
TT	151	31	3	2	2	3	10	0.30	0.20	0.20	0.30	1.30	1
TT	151	33	2	1	5	3	11	0.27	0.45	0.09	0.18	1.18	1
TT	191	1	4	1	9	2	16	0.13	0.56	0.06	0.25	1.25	0
TT	191	2	1	2	9	1	13	0.08	0.69	0.15	0.08	1.08	1

TT	191	3	1	1	8	4	14	0.29	0.57	0.07	0.07	1.07	0
TT	191	4	2	0	9	2	13	0.15	0.69	0.00	0.15	1.15	0
TT	191	5	3	3	7	3	16	0.19	0.44	0.19	0.19	1.19	0
TT	191	6	2	2	5	2	11	0.18	0.45	0.18	0.18	1.18	1
TT	191	8	2	0	6	6	14	0.43	0.43	0.00	0.14	1.14	0
TT	191	9	1	1	11	2	15	0.13	0.73	0.07	0.07	1.07	0
TT	191	13	5	4	5	1	15	0.07	0.33	0.27	0.33	1.33	0
TT	191	14	4	1	8	1	14	0.07	0.57	0.07	0.29	1.29	1
TT	191	15	4	3	5	3	15	0.20	0.33	0.20	0.27	1.27	1
TT	191	16	1	2	5	2	10	0.20	0.50	0.20	0.10	1.10	1
TT	191	19	1	2	6	4	13	0.31	0.46	0.15	0.08	1.08	1
TT	191	22	2	1	9	3	15	0.20	0.60	0.07	0.13	1.13	0
TT	191	25	2	1	5	4	12	0.33	0.42	0.08	0.17	1.17	1
TT	1T-4	4	2	0	6	5	13	0.38	0.46	0.00	0.15	1.15	1
TT	1T-4	5	4	0	8	1	13	0.08	0.62	0.00	0.31	1.31	1
TT	1T-4	6	5	1	6	4	16	0.25	0.38	0.06	0.31	1.31	1
TT	1T-4	1	2	1	6	1	10	0.10	0.60	0.10	0.20	1.20	0
TT	1T-4	9	4	2	8	2	16	0.13	0.50	0.13	0.25	1.25	0
TT	1T-4	10	3	0	5	6	14	0.43	0.36	0.00	0.21	1.21	1
TT	1T-4	11	2	0	7	4	13	0.31	0.54	0.00	0.15	1.15	1
TT	1T-4	14	2	3	7	1	13	0.08	0.54	0.23	0.15	1.15	0
TT	1T-4	12	1	1	7	1	10	0.10	0.70	0.10	0.10	1.10	1
TT	1T-4	17	4	0	4	4	12	0.33	0.33	0.00	0.33	1.33	0
TT	1T-4	13	5	0	10	1	16	0.06	0.63	0.00	0.31	1.31	0
TT	1T-4	15	0	3	4	4	11	0.36	0.36	0.27	0.00	1.00	0
TT	1T-4	16	2	0	6	4	12	0.33	0.50	0.00	0.17	1.17	0
TT	1T-4	19	1	1	8	4	14	0.29	0.57	0.07	0.07	1.07	1
TT	1T-4	20	5	0	4	5	14	0.36	0.29	0.00	0.36	1.36	0
TT	2T-33	3	1	0	7	3	11	0.27	0.64	0.00	0.09	1.09	1
TT	2T-33	4	0	1	10	3	14	0.21	0.71	0.07	0.00	1.00	0
TT	2T-33	5	1	1	7	3	12	0.25	0.58	0.08	0.08	1.08	0
TT	2T-33	6	2	2	6	3	13	0.23	0.46	0.15	0.15	1.15	1
TT	2T-33	7	1	1	6	3	11	0.27	0.55	0.09	0.09	1.09	1
TT	2T-33	8	2	1	4	5	12	0.42	0.33	0.08	0.17	1.17	1
TT	2T-33	9	0	1	6	4	11	0.36	0.55	0.09	0.00	1.00	1
TT	2T-33	10	1	0	4	5	10	0.50	0.40	0.00	0.10	1.10	1
TT	2T-33	11	2	3	4	1	10	0.10	0.40	0.30	0.20	1.20	1
TT	2T-33	12	1	0	9	1	11	0.09	0.82	0.00	0.09	1.09	0
TT	2T-33	14	3	0	2	5	10	0.50	0.20	0.00	0.30	1.30	1
TT	2T-33	17	2	1	4	3	10	0.30	0.40	0.10	0.20	1.20	1
TT	2T-33	18	3	0	8	2	13	0.15	0.62	0.00	0.23	1.23	0
TT	2T-33	19	3	1	6	2	12	0.17	0.50	0.08	0.25	1.25	1
TT	3T-24	2	2	2	9	0	13	0.00	0.69	0.15	0.15	1.15	1

TT	3T-24	3	2	1	7	4	14	0.29	0.50	0.07	0.14	1.14	0
TT	3T-24	7	0	4	6	4	14	0.29	0.43	0.29	0.00	1.00	1
TT	3T-24	8	4	1	8	3	16	0.19	0.50	0.06	0.25	1.25	0
TT	3T-24	9	2	1	9	1	13	0.08	0.69	0.08	0.15	1.15	0
TT	3T-24	10	1	2	7	5	15	0.33	0.47	0.13	0.07	1.07	1
TT	3T-24	12	2	2	9	1	14	0.07	0.64	0.14	0.14	1.14	0
TT	3T-24	13	0	1	11	2	14	0.14	0.79	0.07	0.00	1.00	1
TT	3T-24	16	5	0	5	4	14	0.29	0.36	0.00	0.36	1.36	0
TT	3T-24	17	2	0	7	3	12	0.25	0.58	0.00	0.17	1.17	0
TT	4T-8	1	2	3	1	4	10	0.40	0.10	0.30	0.20	1.20	1
TT	4T-8	3	2	3	4	1	10	0.10	0.40	0.30	0.20	1.20	0
TT	4T-8	4	3	3	4	3	13	0.23	0.31	0.23	0.23	1.23	0
TT	4T-8	6.1	3	1	5	2	11	0.18	0.45	0.09	0.27	1.27	0
TT	4T-8	6.2	2	1	6	3	12	0.25	0.50	0.08	0.17	1.17	0
TT	4T-8	9	2	0	7	4	13	0.31	0.54	0.00	0.15	1.15	0
TT	4T-8	12	4	0	6	6	16	0.38	0.38	0.00	0.25	1.25	0
TT	4T-8	14	4	1	5	1	11	0.09	0.45	0.09	0.36	1.36	1
TT	4T-8	16	5	1	4	3	13	0.23	0.31	0.08	0.38	1.38	0
TT	4T-8	17	2	1	5	6	14	0.43	0.36	0.07	0.14	1.14	1
TT	4T-8	18	6	0	5	4	15	0.27	0.33	0.00	0.40	1.40	0
TT	4T-8	19	2	1	8	2	13	0.15	0.62	0.08	0.15	1.15	0
TT	4T-8	22	4	2	7	2	15	0.13	0.47	0.13	0.27	1.27	0
TT	4T-8	23	1	1	4	4	10	0.40	0.40	0.10	0.10	1.10	1
TT	4T-8	24	6	1	4	3	14	0.21	0.29	0.07	0.43	1.43	0
TT	6T-24	275	4	1	9	2	16	0.13	0.56	0.06	0.25	1.25	1
TT	6T-24	7	4	0	5	3	12	0.25	0.42	0.00	0.33	1.33	1
TT	6T-24	345	6	0	2	2	10	0.20	0.20	0.00	0.60	1.60	0
TT	6T-24	1	2	1	6	7	16	0.44	0.38	0.06	0.13	1.13	0
TT	6T-24	278	2	1	9	3	15	0.20	0.60	0.07	0.13	1.13	0
TT	6T-24	6	2	1	10	3	16	0.19	0.63	0.06	0.13	1.13	0
TT	6T-24	342	2	0	6	7	15	0.47	0.40	0.00	0.13	1.13	0
TT	6T-24	335	2	1	6	1	10	0.10	0.60	0.10	0.20	1.20	1
TT	6T-24	291	4	0	5	3	12	0.25	0.42	0.00	0.33	1.33	1
TT	6T-24	292	6	0	2	3	11	0.27	0.18	0.00	0.55	1.55	0
TT	6T-24	4	2	0	6	4	12	0.33	0.50	0.00	0.17	1.17	0
TT	6T-24	265	2	1	6	4	13	0.31	0.46	0.08	0.15	1.15	0
TT	6T-24	267	4	0	6	6	16	0.38	0.38	0.00	0.25	1.25	0
TT	6T-24	9	3	0	3	8	14	0.57	0.21	0.00	0.21	1.21	0
TT	6T-24	312	4	1	6	5	16	0.31	0.38	0.06	0.25	1.25	0

Table S4 notes: Primary phenotyping of metaphase spreads from plants segregating ASY1 and ASY3, experiment 1. Gen = genotype at both loci, where: DD = DDDD ASY1, DDDD ASY3; DT = DDDD ASY1, TTTT ASY3; TD = TTTT ASY1, DDDD ASY3; TT = TTTT ASY1, TTTT ASY3. All measures as for Table S1. All images are available at: doi: 10.3929/ethz-b-000386103

Table S5: Cytological trait values for *A. arenosa* tetraploids segregating ASY1 and ASY3 – experiment 2

								Proportions (out of total scorable bivalents)					
**Gen**	**Plant**	**Image**	**O**	**+**	**ł**	**l**	**Ts**	**O**	**+**	**ł**	**l**	**CO/Bv**	**MV**
DD	88-1-A5x63-1-C4-1	2	1	5	4	3	13	0.1	0.38	0.31	0.23	1.08	0
DD	88-1-A5x63-1-C4-1	4	1	1	9	3	14	0.1	0.07	0.64	0.21	1.07	1
DD	88-1-A5x63-1-C4-1	5	1	3	4	4	12	0.1	0.25	0.33	0.33	1.08	1
DD	88-1-A5x63-1-C4-1	6	4	2	5	4	15	0.3	0.13	0.33	0.27	1.27	0
DD	88-1-A5x63-1-C4-1	8	2	2	3	6	13	0.2	0.15	0.23	0.46	1.15	1
DD	88-1-A5x63-1-C4-1	9	0	6	6	3	15	0	0.4	0.4	0.2	1	0
DD	88-1-A5x63-1-C4-1	10	3	2	7	4	16	0.2	0.13	0.44	0.25	1.19	1
DD	88-1-A5x63-1-C4-1	11	4	1	7	3	15	0.3	0.07	0.47	0.2	1.27	1
DD	88-1-A5x63-1-C4-1	12	2	4	7	2	15	0.1	0.27	0.47	0.13	1.13	0
DD	88-1-A5x63-1-C4-1	13	4	5	3	0	12	0.3	0.42	0.25	0	1.33	1
DD	88-1-A5x63-1-C4-4	1	2	4	5	0	11	0.2	0.36	0.45	0	1.18	1
DD	88-1-A5x63-1-C4-4	2	0	5	5	3	13	0	0.38	0.38	0.23	1	1
DD	88-1-A5x63-1-C4-4	3	2	5	3	3	13	0.2	0.38	0.23	0.23	1.15	1
DD	88-1-A5x63-1-C4-4	4	1	4	4	3	12	0.1	0.33	0.33	0.25	1.08	1
DD	88-1-A5x63-1-C4-4	5	0	3	7	6	16	0	0.19	0.44	0.38	1	1
DD	88-1-A5x63-1-C4-4	6	3	3	4	3	13	0.2	0.23	0.31	0.23	1.23	0
DD	88-1-A5x63-1-C4-4	7	1	5	4	1	11	0.1	0.45	0.36	0.09	1.09	1
DD	88-1-A5x63-1-C4-6	1	0	3	5	3	11	0	0.27	0.45	0.27	1	1
DD	88-1-A5x63-1-C4-6	2	3	3	5	2	13	0.2	0.23	0.38	0.15	1.23	1
DD	88-1-A5x63-1-C4-6	3	2	6	3	2	13	0.2	0.46	0.23	0.15	1.15	0
DD	88-1-A5x63-1-C4-6	4	0	4	7	0	11	0	0.36	0.64	0	1	1
DD	88-1-A5x63-1-C4-6	5	1	5	4	2	12	0.1	0.42	0.33	0.17	1.08	1
DD	88-1-A5x63-1-C4-6	6	1	4	7	1	13	0.1	0.31	0.54	0.08	1.08	1
DD	88-1-A5x63-1-C4-6	8	4	4	4	1	13	0.3	0.31	0.31	0.08	1.31	1
DD	88-1-A5x63-1-C4-6	9	0	4	3	1	8	0	0.5	0.38	0.13	1	1
DD	P1-17	55	3	2	3	2	11	0.3	0.18	0.27	0.18	1.36	0
DD	P1-17	93	2	4	4	1	11	0.2	0.36	0.36	0.09	1.18	1
DD	P1-17	107	1	5	6	2	15	0.1	0.33	0.4	0.13	1.13	1
DD	P1-17	115	1	1	5	5	12	0.1	0.08	0.42	0.42	1.08	1
DD	P1-17	116	1	5	6	2	14	0.1	0.36	0.43	0.14	1.07	1
DD	P1-17	117	1	5	6	3	16	0.1	0.31	0.38	0.19	1.13	0
DD	P1-17	120	0	6	6	0	12	0	0.5	0.5	0	1	1
DD	P1-17	126	3	4	2	0	10	0.3	0.4	0.2	0	1.4	1
DD	P1-17	129	6	3	1	2	12	0.5	0.25	0.08	0.17	1.5	1
DD	P1-17	1065	0	2	6	4	12	0	0.17	0.5	0.33	1	1
DT	88-1-D4x88-1-A2-12	1	2	6	5	2	15	0.1	0.4	0.33	0.13	1.13	0
DT	88-1-D4x88-1-A2-12	2	1	5	4	1	11	0.1	0.45	0.36	0.09	1.09	1
DT	88-1-D4x88-1-A2-12	3	3	1	8	0	12	0.3	0.08	0.67	0	1.25	1
DT	88-1-D4x88-1-A2-12	5	4	4	2	4	14	0.3	0.29	0.14	0.29	1.29	1
DT	88-1-D4x88-1-A2-12	6	4	5	2	2	13	0.3	0.38	0.15	0.15	1.31	1

DT	88-1-D4x88-1-A2-12	8	4	2	3	2	11	0.4	0.18	0.27	0.18	1.36	1
DT	88-1-D4x88-1-A2-12	9	1	5	2	3	11	0.1	0.45	0.18	0.27	1.09	0
DT	88-1-D4x88-1-A2-12	10	1	5	5	2	13	0.1	0.38	0.38	0.15	1.08	1
DT	88-1-D4x88-1-A2-2	1	1	3	7	0	11	0.1	0.27	0.64	0	1.09	1
DT	88-1-D4x88-1-A2-2	2	2	2	5	5	14	0.1	0.14	0.36	0.36	1.14	1
DT	88-1-D4x88-1-A2-2	3	3	5	4	2	14	0.2	0.36	0.29	0.14	1.21	0
DT	88-1-D4x88-1-A2-2	4	4	2	1	3	10	0.4	0.2	0.1	0.3	1.4	1
DT	88-1-D4x88-1-A2-2	5	1	4	5	2	12	0.1	0.33	0.42	0.17	1.08	1
DT	88-1-D4x88-1-A2-2	6	3	4	3	3	13	0.2	0.31	0.23	0.23	1.23	1
DT	88-1-D4x88-1-A2-2	7	1	0	8	5	14	0.1	0	0.57	0.36	1.07	1
DT	88-1-D4x88-1-A2-2	8	2	5	3	4	14	0.1	0.36	0.21	0.29	1.14	1
DT	88-1-D4x88-1-A2-6	1	5	2	2	2	11	0.5	0.18	0.18	0.18	1.45	1
DT	88-1-D4x88-1-A2-6	2	6	1	2	3	12	0.5	0.08	0.17	0.25	1.5	1
DT	88-1-D4x88-1-A2-6	4	1	4	6	1	12	0.1	0.33	0.5	0.08	1.08	1
DT	88-1-D4x88-1-A2-6	5	1	3	5	2	11	0.1	0.27	0.45	0.18	1.09	1
DT	88-1-D4x88-1-A2-6	6	0	3	6	5	14	0	0.21	0.43	0.36	1	1
DT	88-1-D4x88-1-A2-6	8	3	0	4	3	10	0.3	0	0.4	0.3	1.3	1
DT	88-1-D4x88-1-A2-7	1	2	1	6	3	12	0.2	0.08	0.5	0.25	1.17	0
DT	88-1-D4x88-1-A2-7	2	3	1	5	3	12	0.3	0.08	0.42	0.25	1.25	0
DT	88-1-D4x88-1-A2-7	3	1	2	2	6	11	0.1	0.18	0.18	0.55	1.09	1
DT	88-1-D4x88-1-A2-7	4	7	2	3	3	15	0.5	0.13	0.2	0.2	1.47	0
DT	88-1-D4x88-1-A2-7	5	1	4	5	0	10	0.1	0.4	0.5	0	1.1	1
DT	88-1-D4x88-1-A2-7	6	3	3	4	4	14	0.2	0.21	0.29	0.29	1.21	1
DT	88-1-D4x88-1-A2-7	7	7	2	5	1	15	0.5	0.13	0.33	0.07	1.47	0
DT	88-1-D4x88-1-A2-7	7	1	4	5	3	13	0.1	0.31	0.38	0.23	1.08	1
DT	88-1-D4x88-1-A2-7	8	1	3	6	2	12	0.1	0.25	0.5	0.17	1.08	0
DT	88-1-D4x88-1-A2-7	9	3	3	5	2	13	0.2	0.23	0.38	0.15	1.23	0
DT	88-1-D4x88-1-A2-9	1	3	1	6	2	12	0.3	0.08	0.5	0.17	1.25	1
DT	88-1-D4x88-1-A2-9	2	3	2	4	3	12	0.3	0.17	0.33	0.25	1.25	1
DT	88-1-D4x88-1-A2-9	3	1	2	4	3	10	0.1	0.2	0.4	0.3	1.1	1
DT	88-1-D4x88-1-A2-9	4	3	2	5	2	12	0.3	0.17	0.42	0.17	1.25	1
DT	88-1-D4x88-1-A2-9	5	3	4	1	3	11	0.3	0.36	0.09	0.27	1.27	1
DT	88-1-D4x88-1-A2-9	6	3	5	2	4	14	0.2	0.36	0.14	0.29	1.21	1
DT	88-1-D4x88-1-A2-9	7	2	5	4	2	13	0.2	0.38	0.31	0.15	1.15	1
TD	88-1-A4x88-1-A6-16	1	4	3	5	0	12	0.3	0.25	0.42	0	1.33	1
TD	88-1-A4x88-1-A6-16	2	1	2	4	6	13	0.1	0.15	0.31	0.46	1.08	1
TD	88-1-A4x88-1-A6-16	3	2	8	3	3	16	0.1	0.5	0.19	0.19	1.13	1
TD	88-1-A4x88-1-A6-16	4	1	6	3	1	11	0.1	0.55	0.27	0.09	1.09	1
TD	88-1-A4x88-1-A6-16	5	3	2	6	3	14	0.2	0.14	0.43	0.21	1.21	0
TD	88-1-A4x88-1-A6-16	6	2	6	2	4	14	0.1	0.43	0.14	0.29	1.14	0
TD	88-1-A4x88-1-A6-16	7	1	4	4	3	12	0.1	0.33	0.33	0.25	1.08	1
TD	88-1-A4x88-1-A6-16	8	2	3	1	4	10	0.2	0.3	0.1	0.4	1.2	1
TD	88-1-A4x88-1-A6-16	9	2	4	2	7	15	0.1	0.27	0.13	0.47	1.13	0

TD	88-1-A4x88-1-A6-3	1	3	2	3	3	11	0.3	0.18	0.27	0.27	1.27	0
TD	88-1-A4x88-1-A6-3	1	1	3	5	2	11	0.1	0.27	0.45	0.18	1.09	1
TD	88-1-A4x88-1-A6-3	2	2	2	5	5	14	0.1	0.14	0.36	0.36	1.14	1
TD	88-1-A4x88-1-A6-3	3	1	2	5	4	12	0.1	0.17	0.42	0.33	1.08	1
TD	88-1-A4x88-1-A6-3	4	3	4	2	5	14	0.2	0.29	0.14	0.36	1.21	0
TD	88-1-A4x88-1-A6-3	5	0	3	8	3	14	0	0.21	0.57	0.21	1	1
TD	88-1-A4x88-1-A6-3	6	2	3	4	3	12	0.2	0.25	0.33	0.25	1.17	0
TD	88-1-A4x88-1-A6-3	8	2	2	2	6	12	0.2	0.17	0.17	0.5	1.17	1
TD	88-1-A4x88-1-A6-7	1	2	5	4	5	16	0.1	0.31	0.25	0.31	1.13	1
TD	88-1-A4x88-1-A6-7	3	1	5	5	1	12	0.1	0.42	0.42	0.08	1.08	0
TD	88-1-A4x88-1-A6-7	4	4	1	2	3	10	0.4	0.1	0.2	0.3	1.4	1
TD	88-1-A4x88-1-A6-7	5	3	4	3	1	11	0.3	0.36	0.27	0.09	1.27	1
TD	88-1-A4x88-1-A6-7	6	0	1	6	5	12	0	0.08	0.5	0.42	1	1
TD	88-1-A4x88-1-A6-7	7	2	4	5	2	13	0.2	0.31	0.38	0.15	1.15	1
TD	88-1-A4x88-1-A6-7	9	1	3	3	6	13	0.1	0.23	0.23	0.46	1.08	0
TT	88-1-A2x88-2-A3-10	1	0	4	5	5	14	0	0.29	0.36	0.36	1	0
TT	88-1-A2x88-2-A3-10	2	0	2	4	4	10	0	0.2	0.4	0.4	1	0
TT	88-1-A2x88-2-A3-10	3	0	2	5	4	11	0	0.18	0.45	0.36	1	0
TT	88-1-A2x88-2-A3-10	4	0	1	2	9	12	0	0.08	0.17	0.75	1	1
TT	88-1-A2x88-2-A3-10	5	0	2	8	2	12	0	0.17	0.67	0.17	1	0
TT	88-1-A2x88-2-A3-10	6	1	4	4	4	13	0.1	0.31	0.31	0.31	1.08	1
TT	88-1-A2x88-2-A3-7	1	0	5	3	7	15	0	0.33	0.2	0.47	1	0
TT	88-1-A2x88-2-A3-7	2	2	6	2	2	12	0.2	0.5	0.17	0.17	1.17	1
TT	88-1-A2x88-2-A3-7	3	0	3	7	3	13	0	0.23	0.54	0.23	1	0
TT	88-1-A2x88-2-A3-7	4	3	5	3	3	14	0.2	0.36	0.21	0.21	1.21	1
TT	88-1-A2x88-2-A3-7	5	2	1	6	2	11	0.2	0.09	0.55	0.18	1.18	1
TT	88-1-A2x88-2-A3-7	6	3	0	3	7	13	0.2	0	0.23	0.54	1.23	1
TT	P1-68	1013	2	2	7	3	14	0.1	0.14	0.5	0.21	1.14	1
TT	P1-68	1022	2	0	5	5	12	0.2	0	0.42	0.42	1.17	0
TT	P1-68	1142	0	0	8	7	15	0	0	0.53	0.47	1	0
TT	P1-68	1154	3	1	2	5	11	0.3	0.09	0.18	0.45	1.27	0
TT	P1-68	1174	1	1	8	4	14	0.1	0.07	0.57	0.29	1.07	0
TT	P1-68	1178	4	1	2	2	10	0.4	0.1	0.2	0.2	1.5	1
TT	P1-68	1184	3	1	6	3	13	0.2	0.08	0.46	0.23	1.23	0
TT	P1-68	1185	4	0	3	5	12	0.3	0	0.25	0.42	1.33	1
TT	P1-68	1195	2	2	4	5	13	0.2	0.15	0.31	0.38	1.15	0
TT	P1-68	1204	3	2	1	6	12	0.3	0.17	0.08	0.5	1.25	1

Table S5 notes: Primary phenotyping of metaphase spreads from plants segregating ASY1 and ASY3, experiment 2. Gen = genotype at both loci, where: DD = DDDD ASY1, DDDD ASY3; DT = DDDD ASY1, TTTT ASY3; TD = TTTT ASY1, DDDD ASY3; TT = TTTT ASY1, TTTT ASY3. All measures as for Table S1. All images are available at: doi: 10.3929/ethz-b-000386103

Table S6: Chi-squared test results for bivalent shape comparisons of tetraploids segregating D vs T alleles of ASY1 and ASY3

**Comparison**	**p-value**
All Genotypes	5.82×10^{-16}
**Comparison**	**Bonferroni adjusted p-value**
DD vs. DT	5.98×10^{-03}
DD vs. TD	4.75×10^{-05}
DD vs. TT	4.60×10^{-15}
DT vs. TD	0.167
DT vs. TT	8.22×10^{-09}
TD vs. TT	8.94×10^{-03}

Chi-squared test results for MV rates

**Comparison**	**p-value**
All Genotypes	0.318
**Fishers test comparison**	**Bonferroni adjusted p-value**
DD vs. DT	1
DD vs. TD	1
DD vs. TT	0.624
DT vs. TD	1
DT vs. TT	1
TD vs. TT	1

Table S6 notes: Results from post-hoc pairwise chi-squared tests with Bonferroni correction to determine if significant differences occurred between individual pairs of genotypes using bivalent count data from Table S1. For multivalent presence/absence data we used a Chi-squared test for the entire dataset and a Fishers exact test to compare all genotypes pairwise.

Table S7: Estimated trait means (number per cell) of different bivalent shapes and p-values from GLMMs for ASY1 and ASY3 D vs T

Rod			
	Est mean	2.5% Conf	97.5% Conf
DD (7 plants, 73 cells)	2.2	1.82	2.66
DT (10 plants, 114 cells)	2.29	1.97	2.67
TD (9 plants, 105 cells)	2.7	2.32	3.14
TT (10 plants, 121 cells)	3.29	2.88	3.76

Rod p values			
	DD	DT	TD
DD			
DT	0.751		
TD	0.101	0.136	
TT	0.0007	0.00047	0.0541

Cross			
	Est mean	2.5% Conf	97.5% Conf
DD (7 plants, 73 cells)	2.59	1.81	3.71
DT (10 plants, 114 cells)	2.06	1.52	2.79
TD (9 plants, 105 cells)	1.65	1.19	2.29
TT (10 plants, 121 cells)	1.25	0.91	1.72

Cross p values			
	DD	DT	TD
DD			
DT	0.342		
TD	0.0677	0.325	
TT	0.0029	0.0255	0.234

Ring			
	Est mean	2.5% Conf	97.5% Conf
DD (7 plants, 73 cells)	1.95	1.54	2.45
DT (10 plants, 114 cells)	2.62	2.2	3.13
TD (9 plants, 105 cells)	2.32	1.91	2.81
TT (10 plants, 121 cells)	2.31	1.92	2.78

Ring p values			
	DD	DT	TD
DD			
DT	0.0442		
TD	0.2499	0.3549	
TT	0.251	0.3266	0.976

Bowtie			
	Est mean	2.5% Conf	97.5% Conf
DD (7 plants, 73 cells)	5.53	4.69	6.52
DT (10 plants, 114 cells)	5.44	4.74	6.24
TD (9 plants, 105 cells)	5.81	5.05	6.7
TT (10 plants, 121 cells)	5.67	4.96	6.48

Bowtie p values			
	DD	DT	TD
DD			
DT	0.88		
TD	0.652	0.508	
TT	0.821	0.674	0.798

MV			
	Est mean	2.5% Conf	97.5% Conf
DD (7 plants, 73 cells)	0.61	0.447	0.751
DT (10 plants, 114 cells)	0.588	0.452	0.712
TD (9 plants, 105 cells)	0.554	0.417	0.684
TT (10 plants, 121 cells)	0.461	0.337	0.59

Multivalent p values			
	DD	DT	TD
DD			
DT	0.836		
TD	0.601	0.725	
TT	0.159	0.183	0.335

Table S7 notes: Trait means (left) and p-values (right) calculated from GLMM modeling. Normal approximations of 95% confidence intervals (range given from 2.5% Conf to 97.5% Conf) were calculated as 1.96 estimated standard errors.

Table S8: Quantitative measures from prophase I immunocytology of ASY1 and ASY3 segregants

	Mean	TT	DT	TD	DD
**Total SC per cell (microns)**	**Mean of all measurements**	**478.74**	**539.36**	**503.99**	**502.42**
		n=52 s.d.±(47.93784)	n=45 s.d.±(48.2761)	n=34 s.d.±(38.6811)	n=20 s.d.±(58.1761)
	**Replicate 1**	**506.66**	**520.47**	**520.76**	**487.56**
		n=12 s.d.±(34.9381)	n=13 s.d.±(46.2886)	n=10 s.d.±(29.7365)	n=8 s.d.±(48.4238)
	**Replicate 2**	**509.96**	**509.58**	**471.37**	**500.77**
		n=9 s.d.±(52.6656)	n=9 s.d.±(34.5520)	n=3 s.d.±(4.4906)	n=12 s.d.±(63.9219)
	**Replicate 3**	**492.18**	**564.11**	**458.24**	**N/A**
		n=9 s.d.±(34.0380)	n=13 s.d.±(42.2741)	n=8 s.d.±(25.7670)	N/A
	**Replicate 4**	**452.50**	**558.54**	**526.76**	**N/A**
		n=11 s.d.±(31.5146)	n=10 s.d.±(48.9661)	n=13 s.d.±(24.5601)	N/A
	**Replicate 5**	**437.98**			
		n=11 s.d.±(40.9631)			
	**unweighted mean of replicate means**	**479.86**	**538.17**	**494.28**	**500.77**
		±(32.7060)	±(27.1919)	±(34.5445)	±(17.5135)
	**Nested ANOVA estimated mean**	**479.62**	**538.40**	**496.93**	**502.54**
		s.e.±(13.6113)	s.e.±(15.1288)	s.e.±(15.8562)	s.e.±(21.6416)
**Total HEI10 foci per cell**	**Mean of all measurements**	**19.02**	**21.89**	**20.59**	**18.40**
		n=52 s.d.±(2.8179)	n=45 s.d.±(3.8801)	n=34 s.d.±(3.4034)	n=20 s.d.±(2.4794)
	**Replicate 1**	**18.00**	**18.23**	**21.40**	**18.00**
		n=12 s.d.±(3.0451)	n=13 s.d.±(1.5359)	n=10 s.d.±(3.2042)	n=8 s.d.±(1.3093)
	**Replicate 2**	**19.89**	**20.56**	**22.67**	**18.67**
		n=9 s.d.±(2.7131)	n=9 s.d.±(2.0069)	n=3 s.d.±(4.7258)	n=12 s.d.±(3.0551)
	**Replicate 3**	**20.67**	**23.92**	**21.13**	**N/A**
		n=9 s.d.±(3.0822)	n=13 s.d.±(4.0715)	n=8 s.d.±(3.8707)	N/A
	**Replicate 4**	**19.82**	**25.20**	**19.15**	**N/A**
		n=11 s.d.±(2.6389)	n=10 s.d.±(2.3476)	n=13 s.d.±(2.7642)	N/A
	**Replicate 5**	**17.27**			
		n=11 s.d.±(1.1909)			
	**unweighted mean of replicate means**	**19.13**	**21.98**	**21.09**	**18.36**
		±(1.4263)	±(3.1744)	±(1.4528)	±(0.0471)
	**Nested ANOVA estimated mean**	**19.11**	**21.96**	**20.93**	**18.35**
		s.e.±(0.9139948)	s.e.±(1.0156578)	s.e.±(1.0659909)	s.e.±(1.4535158)
**Total SPS sites per cell**	**Mean of all measurements**	**5.13**	**6.98**	**6.09**	**5.75**
		n=52 s.d.±(1.5085)	n=45 s.d.±(1.9828)	n=34 s.d.±(2.6442)	n=20 s.d.±(2.3368)
	**Replicate 1**	**5.58**	**6.92**	**7.40**	**5.75**
		n=12 s.d.±(1.8320)	n=13 s.d.±(1.9348)	n=10 s.d.±(2.1187)	n=8 s.d.±(1.6690)

	**Replicate 2**	**5.11**	**7.00**	**5.67**	**5.75**
		n=9 s.d.±(0.9280)	n=9 s.d.±(1.8708)	n=3 s.d.±(1.1547)	n=12 s.d.±(2.7675)
	**Replicate 3**	**5.11**	**7.08**	**5.13**	**N/A**
		n=9 s.d.±(1.4530)	n=13 s.d.±(2.4987)	n=8 s.d.±(2.1671)	N/A
	**Replicate 4**	**5.18**	**6.90**	**5.77**	**N/A**
		n=11 s.d.±(1.6624)	n=10 s.d.±(1.6633)	n=13 s.d.±(3.2699)	N/A
	**Replicate 5**	**4.64**			
		n=11 s.d.±(1.5015)			
	**unweighted mean of replicate means**	**5.12**	**6.98**	**5.99**	**5.75**
		±(0.3363)	±(0.0803)	±(0.9814)	±(0.0000)
	**Nested ANOVA estimated mean**	**5.13**	**6.98**	**6.09**	**5.75**
		s.e.±(0.2856045)	s.e.±(0.3070157)	s.e.±(0.3532054)	s.e.±(0.4605235)
**Proportional distance of single HEI10 focus from chromosome end**	**Mean of all measurements**	**0.30**	**0.30**	**0.31**	**0.30**
		n=620 s.d.±(0.1381)	n=403 s.d.±(0.1384)	n=345 s.d.±(0.1381)	n=235 s.d.±(0.1434)
	**Replicate 1**	**0.32**	**0.28**	**0.32**	**0.30**
		n=150 s.d.±(0.1307)	n=164 s.d.±(0.1545)	n=91 s.d.±(0.1175)	n=98 s.d.±(0.1416)
	**Replicate 2**	**0.27**	**0.31**	**0.34**	**0.29**
		n=102 s.d.±(0.1317)	n=91 s.d.±(0.1310)	n=23 s.d.±(0.1401)	n=137 s.d.±(0.1452)
	**Replicate 3**	**0.31**	**0.32**	**0.32**	**N/A**
		n=90 s.d.±(0.1424)	n=90 s.d.±(0.1192)	n=81 s.d.±(0.1489)	N/A
**Physical distance of single HEI10 focus from chromosome end**	**Mean of all measurements**	**8.9852**	**9.7775**	**9.5891**	**9.1639**
		n=620 s.d.±(4.5158)	n=403 s.d.±(4.9554)	n=345 s.d.±(4.9163)	n=235 s.d.±(4.9434)
	**Replicate 1**	**10.0662**	**8.8229**	**10.4621**	**9.1031**
		n=150 s.d.±(4.5646)	n=164 s.d.±(5.3048)	n=91 s.d.±(4.6348)	n=98 s.d.±(4.9191)
	**Replicate 2**	**8.4874**	**9.7196**	**10.4431**	**9.2073**
		n=102 s.d.±(4.5862)	n=91 s.d.±(4.7533)	n=23 s.d.±(4.7404)	n=137 s.d.±(4.9783)
	**Replicate 3**	**9.1695**	**11.0840**	**8.6386**	N/A
		n=90 s.d.±(4.5787)	n=90 s.d.±(4.7811)	n=81 s.d.±(4.4868)	N/A
	**Replicate 4**	9.0572	10.5399	9.4419	N/A
		n=129 s.d.±(4.3372)	n=58 s.d.±(3.9280)	n=150 s.d.±(5.2543)	N/A
	**Replicate 5**	**8.0639**			
		n=149 s.d.±(4.3413)			
	**unweighted mean of replicate means**	8.9689	10.0416	9.7464	9.1552
		±(0.7588)	±(0.9872)	±(0.8789)	±(0.0737)

Table S8 notes: Replicates are different individual plants of each genotype (each shown in a different color). n = number of cells measured per individual (or pairs of chromosomes per individual for physical distance of HEI10 foci from nearest chromosomes end). s.d. = standard deviation from the mean.

Plants sampled are as follows (in order as listed in table above):
TT: 2T-33, 1T-4, 4T-3, 4T-18, 191 (2T-33, 1T-4, 191 were also sampled for metaphase)
DT: 1T-5, 3T-31, 4T-13, 4T-15 (4T-15 was also sampled for metaphase)
TD: 1T-17, 2T-25, 143, 171 (1T-17, 2T-25, 143 also sampled for metaphase)
DD: 1T-14, 1T-36 (1T-14 also sampled for metaphase)

References

1. P. Monnahan *et al.*, Pervasive population genomic consequences of genome duplication in Arabidopsis arenosa. *Nat Ecol Evol* **3**, 457-468 (2019).
2. K. M. Wright *et al.*, Selection on meiosis genes in diploid and tetraploid Arabidopsis arenosa. *Mol Biol Evol* **32**, 944-955 (2015).
